# Laminin 211 inhibits protein kinase A in Schwann cells to modulate neuregulin 1 type III-driven myelination

**DOI:** 10.1371/journal.pbio.2001408

**Published:** 2017-06-21

**Authors:** Monica Ghidinelli, Yannick Poitelon, Yoon Kyoung Shin, Dominique Ameroso, Courtney Williamson, Cinzia Ferri, Marta Pellegatta, Kevin Espino, Amit Mogha, Kelly Monk, Paola Podini, Carla Taveggia, Klaus-Armin Nave, Lawrence Wrabetz, Hwan Tae Park, Maria Laura Feltri

**Affiliations:** 1 Hunter James Kelly Research Institute, Department of Biochemistry and Neurology, Jacobs School of Medicine and Biomedical Sciences, The State University of New York at Buffalo, Buffalo, New York, United States of America; 2 Division of Genetics and Cell Biology, San Raffaele Scientific Institute, DIBIT, Milano, Italy; 3 UniSR, Vita Salute San Raffaele University, Milan, Italy; 4 Department of Physiology, Peripheral Neuropathy Research Center, Dong-A University Medical School, Busan, South Korea; 5 Department of Developmental Biology, Washington University School of Medicine, St. Louis, Missouri, United States of America; 6 Division of Neuroscience and INSPE, San Raffaele Scientific Institute, DIBIT, Milano, Italy; 7 Department of Neurogenetics, Max Planck Institute of Experimental Medicine, Göttingen, Germany; Oregon Health and Science University, United States of America

## Abstract

Myelin is required for proper nervous system function. Schwann cells in developing nerves depend on extrinsic signals from the axon and from the extracellular matrix to first sort and ensheathe a single axon and then myelinate it. Neuregulin 1 type III (Nrg1III) and laminin α2β1γ1 (Lm211) are the key axonal and matrix signals, respectively, but how their signaling is integrated and if each molecule controls both axonal sorting and myelination is unclear. Here, we use a series of epistasis experiments to show that Lm211 modulates neuregulin signaling to ensure the correct timing and amount of myelination. Lm211 can inhibit Nrg1III by limiting protein kinase A (PKA) activation, which is required to initiate myelination. We provide evidence that excessive PKA activation amplifies promyelinating signals downstream of neuregulin, including direct activation of the neuregulin receptor ErbB2 and its effector Grb2-Associated Binder-1 (Gab1), thereby elevating the expression of the key transcription factors Oct6 and early growth response protein 2 (Egr2). The inhibitory effect of Lm211 is seen only in fibers of small caliber. These data may explain why hereditary neuropathies associated with decreased laminin function are characterized by focally thick and redundant myelin.

## Author summary

Myelin is formed by the wrapping of glial cell membranes around axons and is required for the fast conduction of nerve impulses and to support axons. In the peripheral nervous system, myelin is produced by Schwann cells. Peripheral myelin defects cause debilitating diseases, whose molecular pathogeneses are only partially understood. Here, we reveal for the first time how 2 crucial extracellular modulators of myelin formation, neuregulin 1 type III (Nrg1III) and laminin α2β1γ1 (Lm211), work together in the peripheral nervous system. Although Lm211 was believed to promote myelination, we show that it can also inhibit myelin formation by suppressing the activity of Nrg1III, limiting the activation of its downstream signaling cascade. These results help to explain why certain inherited neuropathies are characterized by hypermyelination and redundant myelin sheaths.

## Introduction

Myelin is essential for rapid impulse propagation and the proper function of the nervous system. Schwann cells (SCs) form myelin in peripheral nerves in 2 subsequent steps, radial sorting of axons and myelination. During radial sorting, immature SCs segregate axons with a diameter larger than 1 μm to the edge of embryonic axon bundles and then acquire a 1:1 relationship with these axons and differentiate into promyelinating SCs. Immature SCs express the transcription factor Oct6 (Pou3f1) that is later downregulated [1,2], while promyelinating SCs express the transcription factor early growth response protein 2 (Egr2 or Krox20) that is necessary to transition into wrapping and myelination [3]. A signaling pathway consisting of 3′-5′-cyclic adenosine monophosphate (cAMP) and protein kinase A (PKA), possibly via regulation of Nfkb and Oct6, is required to achieve full Egr2 activation and myelination [2,4–7]. Egr2, in turn, is the master regulator of myelin protein and lipid genes [reviewed in 8]. Following Egr2 activation, myelin-forming SCs start to elaborate a myelin sheath around axons. Myelin thickness depends on the number of myelin wraps that a SC makes around an axon and is correlated to axon diameter [9]. Signaling molecules that include Disc Large MAGUK scaffold protein 1 (DLG1) and Phosphatase and Tensin homolog (PTEN) are then required to terminate wrapping [10–12]. Finally, groups of small axons that are not radially sorted into a 1:1 ratio remain ensheathed by nonmyelinating SCs, which organize the associated axons into a Remak bundle.

These developmental steps are regulated by the axonal growth factor neuregulin 1 type III (Nrg1III) and by the extracellular matrix (ECM) component laminin α2β1γ1 (Lm211). However, how these 2 signals are integrated is unknown. Laminins in the basal lamina are required for radial sorting by enabling cytoskeletal rearrangements needed for changes in SC morphology [13,14]. The major SC laminin is composed of α2, β1, and γ1 chains, encoded by *Lama2*, *Lamb1*, and *Lamc1* genes, respectively. Mutations or targeted inactivation of *Lama2* result in radial sorting defects [13,15,16], as does inactivation of the genes encoding the Lm211 receptors a6β1, a7β1 integrins, and dystroglycan [17–19].

Whether Lm211 also controls the initiation of myelin wrapping after radial sorting is unclear. Experiments in vitro indicate that Lm211 promotes the initiation of myelination [20,21], but this could simply represent Lm211 enabling the prerequisite step of radial sorting. *Lama2* deletion in vivo does not prevent myelination, but this could be explained by compensation by other laminins that do not contain the α2 chain [16,22]. In addition, it has been reported that Lm211 positively regulates myelin thickness [23], but in contrast with this finding, laminin signaling via the α6β4 integrin receptor was recently shown to inhibit myelination via serum and glucocorticoid-induced kinase 1 [24], and human biopsies from patients with *LAMA*2 mutations show thickened and irregularly folded myelin sheaths [25,26]. Thus, the role of Lm211 in myelination remains unresolved. On the other hand, it is well established that the level of axonal Nrg1III is a key instructive signal for myelination [27,28] and regulates myelin thickness [27–29]. Nrg1III promotes myelination by engaging the ErbB2/ErbB3 receptor tyrosine kinase, which in turn stimulates several signaling pathways, including PI3K/Akt, Calcineurin and MAPK/ERK, which are thought to converge on Oct6 and Egr2 activation. Although Nrg1III has a clear role in the initiation of myelination, its role during axonal sorting has not been proven directly. A role for Nrg1III in radial sorting is suggested by the observation that SCs fail to ensheathe Nrg1III-deficient axons in vitro and by the fact that *Nrg1III* haploinsufficiency causes defects in axon ensheathment in Remak bundles [27,30]. Thus, the role that *Nrg1III* plays during axonal sorting is unclear. Finally, how Nrg1III signaling from axons is integrated with Lm211 signaling from the basal lamina is poorly understood.

Here, using genetic epistasis experiments in vivo and in vitro, we show that Lm211 and Nrg1III interact functionally. By modulating the expression of Nrg1III and Lm211, we show that in early development and in small fibers, Lm211 inhibits Nrg1III-driven myelination, but this effect is only revealed when the amount of Nrg1III is also altered. How does Lm211 inhibit Nrg1III? cAMP and PKA are required in parallel to Nrg1III to initiate myelination, by functioning as a switch that triggers Egr2 expression, but cAMP/PKA are not required for Egr2 maintenance [4,5,31]. Here, we show that Lm211 inhibits PKA activity and various signaling steps downstream of Nrg1III. We propose that Lm211 limits Nrg1III signaling via PKA to prevent precocious myelination during radial sorting, inappropriate myelination of small fibers that normally do not become myelinated, and excessive myelin wrapping of small myelinated fibers.

## Results

### SC primed by Lm211 requires Nrg1III to myelinate in vitro

**Fig 1:**
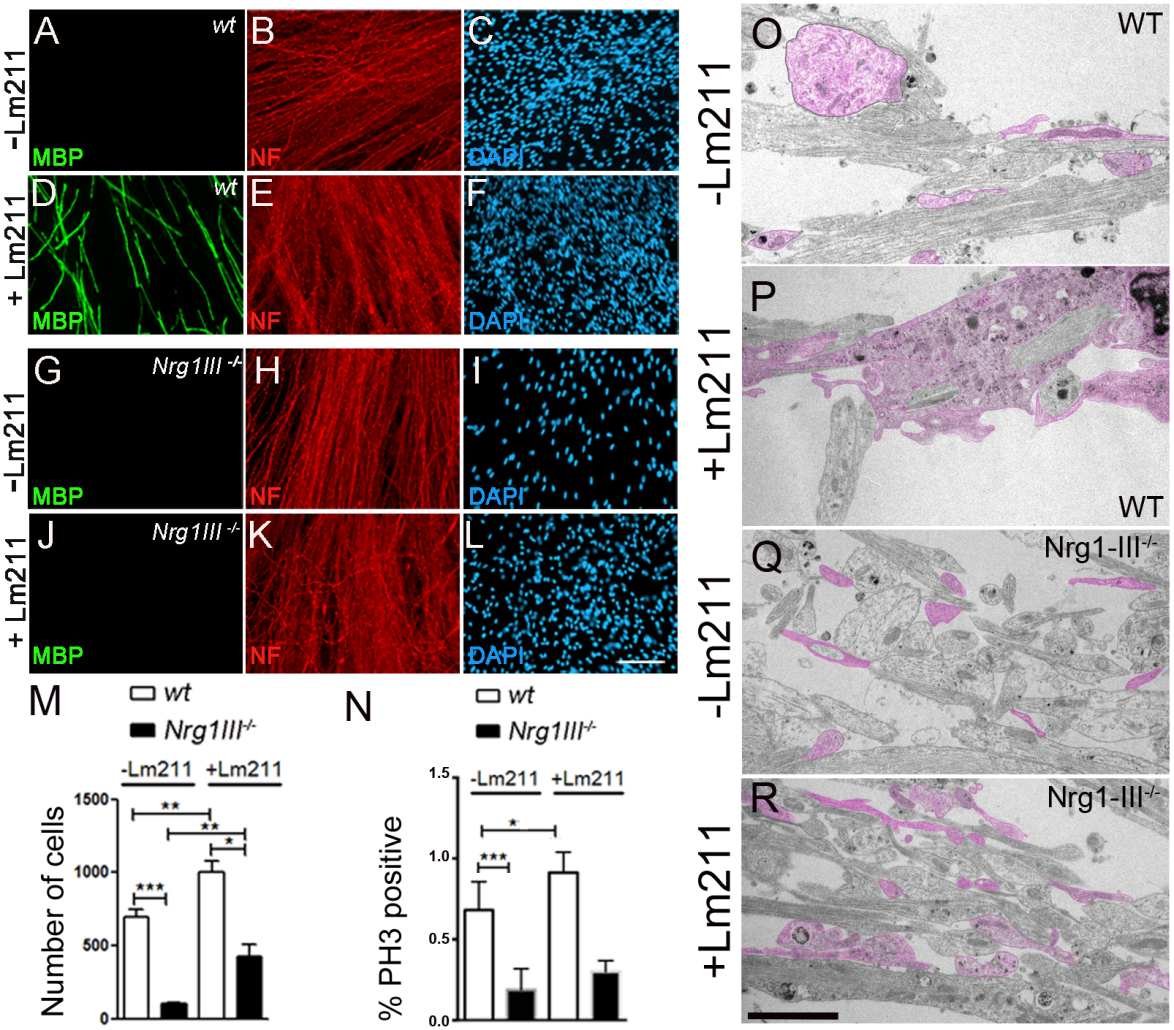
Laminin α2β1γ1 (Lm211) leads to myelination only in the presence of neuregulin 1 type III (Nrg1III). Wild-type (WT) rat Schwann cells (SCs) cocultured with dorsal root ganglia (DRG) neurons from WT (A-F) or *Nrg1III*^*−/−*^ (G-L) mouse embryos were maintained in media with or without Lm211 (50 μg/mL), fixed and stained for myelin basic protein (MBP) (green), neurofilament (red), and DAPI (blue). Lm211 induces myelination only when SCs contact *Nrg1III*^*wt*^ neurons. (M) Quantification of the number of SCs per field of view. (N) Number of phosphorylated-histone3 (P-H3) positive nuclei in the cultures. Images are representative of 3 independent experiments. *n* = 6 coverslips for each treatment. (O-R) Electron micrographs of the cultures show that Lm211 promotes ensheathment of WT axons by SC processes (pseudocolored in pink). *Nrg1III*^*−/−*^axons are not ensheathed, but Lm211 improves the intermingling of SC and their processes (pseudocolored in pink) parallel to axons. Bar = 50 μm in L; 2,5 μm in O. The numerical data used in M–N are included in S1 Data.

It is known that laminins in the SC basal lamina are necessary for radial sorting [32–35], that Lm211 promotes myelination in vitro [21], and can even replace ascorbic acid to induce myelination [20]. While this has been interpreted as Lm211 promoting wrapping and myelination, Lm211 may instead only enable SCs to arrive into a 1:1 relationship with axons (promyelinating stage), which then leads to myelination upon Nrg1 activation independently of laminins. To begin to make this distinction, we first asked if Lm211 could lead to myelination without Nrg1III. We took advantage of the ability of Lm211 to replace ascorbic acid in inducing myelination in vitro and asked if wild-type SCs treated with Lm211 could myelinate *Nrg1III-*deficient neurons. As reported [20], SCs myelinate wild-type neurons when exogenous laminin is added to the media (Fig 1D), while no myelination was present without the addition of Lm211 (Fig 1A). However, Lm211 cannot induce myelination in the absence of Nrg1III (Fig 1J). Fewer SCs were observed in culture with Nrg1III deficient axons, but Lm211 increased the number of SCs (Fig 1M). This was not accompanied by an increase in SC proliferation (Fig 1N) but could be due to improved association of SC with *Nrg1III-*deficient neurons (Fig 1O–1R). This experiment shows that Lm211 signals need to converge with those activated by Nrg1III for myelination to proceed in vitro.

### Nrg1III haploinsufficiency does not exacerbate the aberrant interactions between Lm211-null SCs and axons

**Fig 2:**
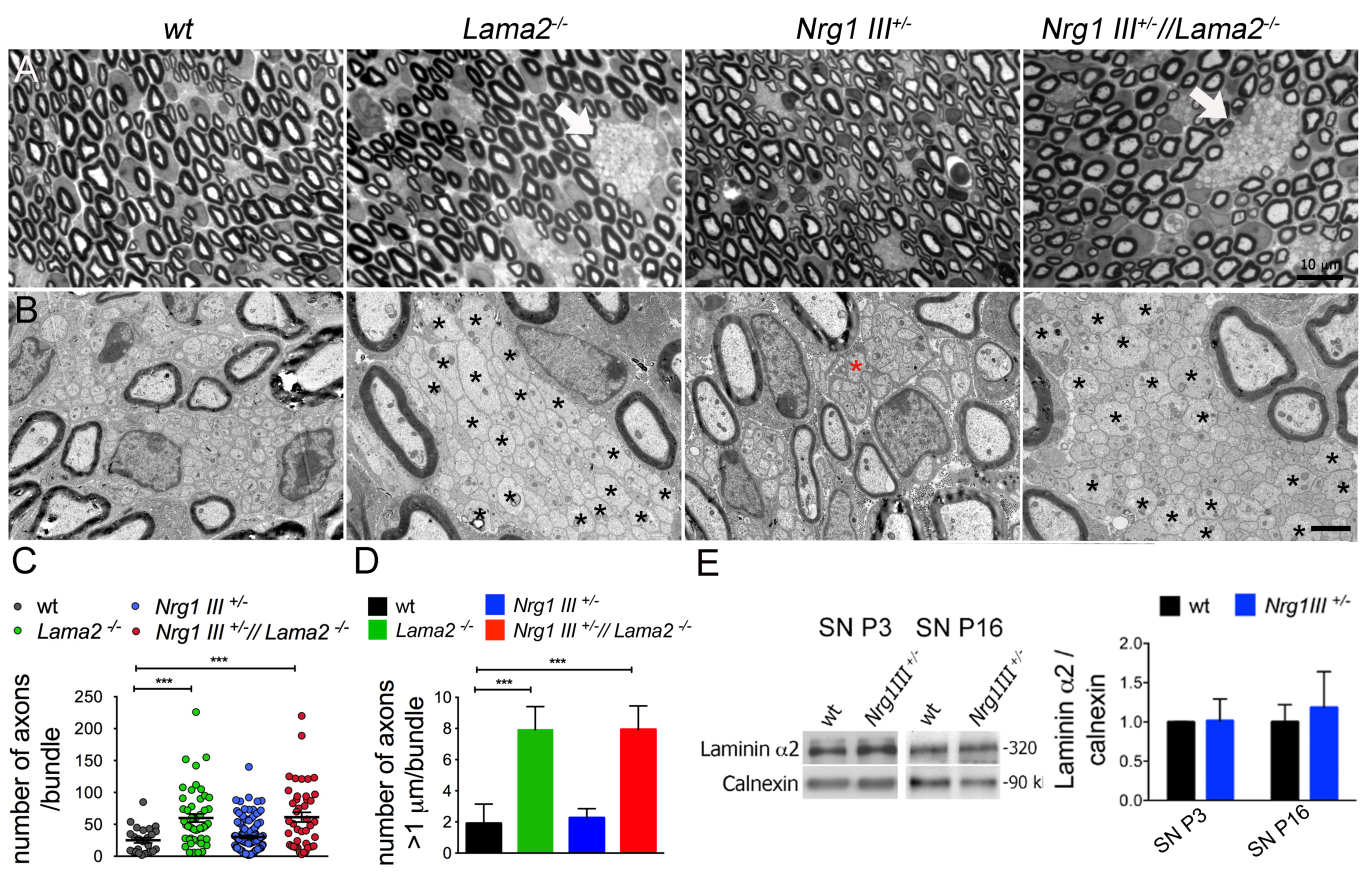
Partial loss of neuregulin 1 type III (Nrg1III) does not impair axonal sorting and does not worsen the axonal sorting defects of laminin α2β1γ1 (Lm211)-deficient mice. (A) Transverse semithin sections of sciatic nerves from the indicated genotypes at postnatal day 16 (P16). *Lama2*^*−/−*^ and *Nrg1III*^*+/−*^*//Lama2*^*−/−*^ mice present with bundles of naked axons, which indicate defective radial sorting (arrows). (B) Electron micrograph analysis shows unsorted bundles that contain numerous amyelinated axons with diameter >1 μm (asterisks) in *Lama2*^*−/−*^ and *Nrg1III*^*+/−*^*//Lama2*^*−/−*^ mice. Nrg1 III^+/−^ nerves do not contain unsorted axon bundles, but only defective Remak fibers (red asterisk). (C) Quantification of the number of axons contained in each bundle at P16. (D) Quantification of the number of amyelinated axons with diameter >1μm per bundle in sciatic nerves at P16. The number of axons >1 μm is increased in *Lama2*^*−/−*^ *mice* (*Lama2*^*−/−*^ 7.91% ± 2.5 versus wild-type (WT) 2.86% ± 1.9); but not in *Nrg1III*^+/−^ mice (*Nrg1III*^+/−^ 2.26 ± 1.0 versus WT 2.86% ± 1.9); in the *Nrg1III*^*+/−*^*//Lama2*^*−/−*^, the percentage of unsorted axons is comparable to *Lama2*^*−/−*^ mice (*Lama2*^*−/−*^ 7.91% ± 2.5 versus *Nrg1III*^+/−^//*Lama2*^*−/−*^ 7.93 ± 2.63). *n* = 3 mice per genotype; 1-way ANOVA with Bonferroni posthoc test for individual comparisons. (E) Western blot analysis shows that the levels of the α2 chain of Lm211 are not decreased in sciatic nerves of *Nrg1III*^*+/−*^ mice. Data are represented as mean value ± SD ***p ≤ 0.001, *n* = 3 mice per genotype; Student *t* test. Bar = 10μm in A, 2 μm in B. The numerical data used in C, D, and E are included in S1 Data.

Myelination is preceded by radial sorting. While it is evident from the literature that Lm211 mutants have impaired radial sorting, a role for Nrg1III in this process is unclear. To test if Lm211 and Nrg1III cooperate to regulate radial sorting, we performed a genetic interaction experiment and asked if partial loss of *Nrg1III* (*Nrg1III* heterozygous null mice, as constitutive nulls are embryonic lethal) worsen the radial sorting defects of *Lama2*^−/−^ mice. We quantified radial sorting defects in sciatic nerves at postnatal day 16 (P16) because *Lama2*^−/−^ mice in the C57/BL6 background die around P21. At P16, radial sorting is completed in normal sciatic nerves, all axons larger than 1 μm have been myelinated, and small bundles, hardly visible by semithin sections (Fig 2A), contain small caliber axons that are beginning to differentiate into mature, nonmyelinated Remak fibers. By electron microscopy (EM), the majority of axonal bundles in wild-type nerves contained fewer than 50 axons that were usually smaller than 1 μm (Fig 2B, 2C and 2D). As previously reported, *Lama2*^−/−^ sciatic nerve presents sizable bundles of axons visible by semithin sections (Fig 2A, arrow), which contain large numbers of naked axons including those with diameters >1 μm (Fig 2B, asterisks, Fig 2C and 2D), a sign of impaired axonal sorting. *Nrg1III*^+/−^ nerves contained abnormally ensheathed, small bundles of axons (Fig 2B, red asterisk), as previously described [27], but no significant increase in the number of naked axons per bundle (Fig 2B and 2C) or in the number of axons with diameter >1 μm in bundles (Fig 2D). Importantly, *Nrg1III* haploinsufficiency did not increase the percentage of unsorted axons in *Nrg1III*^+/−^//*Lama2*^−/−^ nerves (Fig 2). Finally, the levels of laminin α2 protein were normal in *Nrg1III*^+/−^ sciatic nerve at P3 and P16 (Fig 2E), indicating that Nrg1III does not regulate Lm211 expression.

We conclude that the partial loss of Nrg1III in vivo does not cause significant defects in radial sorting nor does it further aggravate radial sorting phenotypes due to loss of Lm211.

### Lm211 inhibits Nrg1III-induced myelination of small fibers

**Fig 3:**
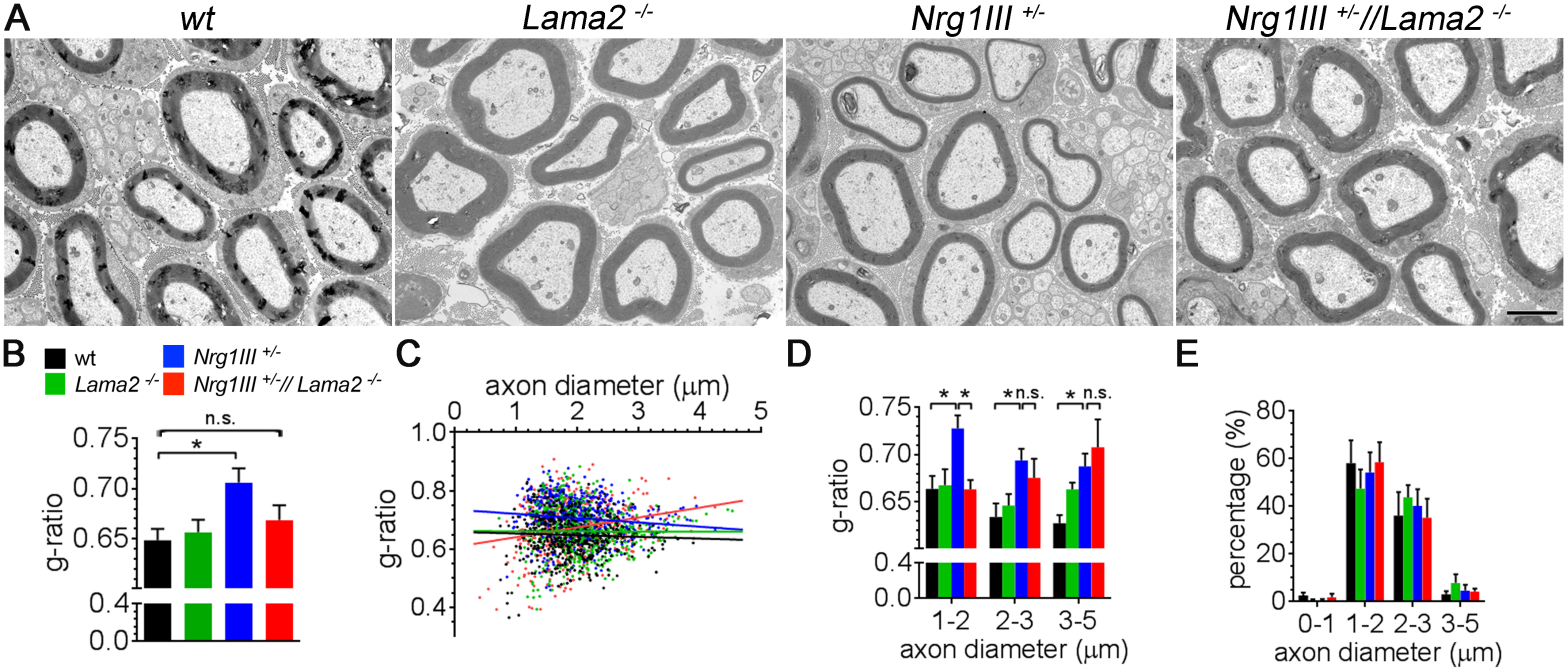
Loss of laminin α2β1γ1 (Lm211) does not affect myelin thickness, but rescues hypomyelination due to neuregulin 1 type III (Nrg1III) haploinsufficiency. (A) Electron micrographs of sciatic nerves from mice of the indicated genotypes at postnatal day 16 (P16). (B) Quantification of g-ratios. At least 150 axons per animal were quantified for 3 animals/genotype, *p < 0.05 by 1-way ANOVA multiple comparison test. (C) Scatter plot displays and distribution (D) of g-ratios of individual fibers as a function of axon diameter shows that in the double mutant, the rescue in myelin thickness mainly occurs on axons with diameters smaller than 2μm. (E) Distribution of diameter of myelinated axons. Bar = 2 μm. The numerical data used in B-E are included in S1 Data.

We next asked if Lm211 and Nrg1III interacted genetically at later timepoints, i.e., during myelination, using the same animals. Axonal Nrg1III levels regulate myelin thickness: *Nrg1III* haploinsufficiency causes hypomyelination (thin myelin) while Nrg1III overexpression causes hypermyelination (thick myelin) [28]. In contrast, the role of laminin in regulating the onset or extent of myelination is unclear. Based on our initial hypothesis that Lm211 and Nrg1III are both promyelinating signals, we asked if loss of *Lama2* further reduced myelination in *Nrg1III*^+/−^ mice. As reported, sciatic nerves from *Nrg1III*^+/−^ mice showed thinner myelin (Fig 3A and 3B). In contrast, sciatic nerves from *Lama*2^−/−^ mice displayed no significant changes in myelin thickness. To our surprise, nerves from *Nrg1III*^+/−^//*Lama*2^−/−^ mice showed a return of myelin thickness close to wild-type levels (Fig 3A and 3B). When the average g-ratio was plotted against the diameter of the fibers, it became clear that the rescue was mainly due to an effect on axons smaller than 2 μm (Fig 3C and 3D). We confirmed that the distribution of axon diameters was not altered in the different genotypes (Fig 3E). This experiment shows that loss of *Lama*2 suppresses the hypomyelination phenotype of *Nrg1III*^+/−^ mice in small fibers. Thus, in a genetic context in which Nrg1III-induced myelination is reduced, the role of Lm211 on myelination becomes evident. These data suggest a repressive role for Lm211 in the myelination of small caliber axons.

### Overexpression of Nrg1III enhances radial sorting defects in *Lama2*^*−/−*^ mice

**Fig 4:**
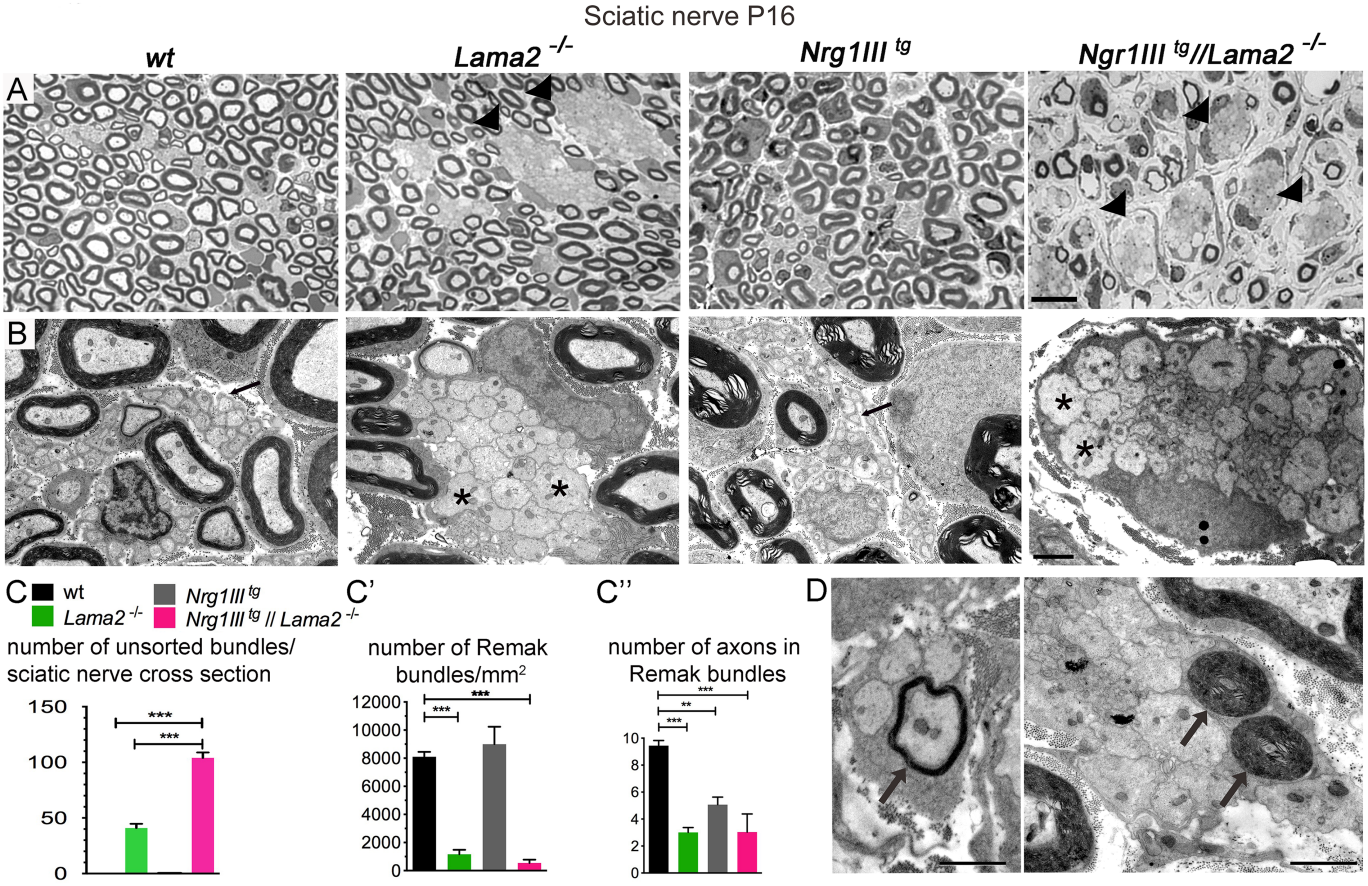
Neuregulin 1 type III (Nrg1III) overexpression worsens the radial sorting defects of Lama2^−/−^ mice. (A) Transverse semithin sections of sciatic nerves from mice of the indicated genotypes at postnatal day 16 (P16) are shown. *Nrg1III*^*tg*^*//Lama2*^*−/−*^ mice have more bundles of naked axons (unsorted bundles) than *Lama2*^−/−^ mice (arrowheads). (B) Electron micrograph analysis shows that in *Lama2*^*−/−*^ and *Nrg1III*^*+/−*^*//Lama2*^*−/−*^ nerves these unsorted bundles contain amyelinated, naked axons with diameter >1 μm (asterisk), while in wild-type (WT) and *Nrg1III*^*tg*^ nerves, there are only Remak bundles that contain axons ensheathed and smaller than 1 μm (arrow). (C) Number of unsorted bundles per nerve cross semithin section, showing a 3-fold increase in the number of unsorted bundles in *Nrg1III*^*tg*^//*Lama*2^−/−^ (106.33 ± 5.7 versus 35.33 ± 0.5 *Lama*2^−/−^ ***p = 0.0005 by Student *t* test; *n* = 3). (C’): The number of Remak bundles on ultrathin electron microscopy (EM) sections is decreased in *Nrg1III*^*tg*^//*Lama*2^−/−^ and *Lama*2^−/−^ mutants (***p = 0.001 by 1-way ANOVA with Bonferroni multiple comparison test, *n* = 4). (C”): All mutant nerves have reduced numbers of axons in Remak bundles on ultrathin EM sections (**p = 0.005; ***p = 0.001 by 1-way ANOVA with Bonferroni multiple comparison test, *n* = 4). (D) Examples of precocious myelination of axons that have not been sorted into a 1:1 relationship (arrows). Bar = 10 μm in A, 2 μm in B and D. The numerical data used in C–C” are included in S1 Data.

So far, we showed that Lm211 may inhibit Nrg1III-induced myelination (Fig 3), although we could not reveal an interaction between the 2 molecules during radial sorting (Fig 2). To further explore these results, we crossed mice that overexpress Nrg1III (*Nrg1III*^*tg*^)[36] with *Lama*2^−/−^ mice and analyzed sciatic nerve morphology at P16 by EM. *Nrg1III*^*tg*^ nerves had normal Remak bundles (arrow in B) and no abnormal unsorted bundles of axons (Fig 4A, 4B and 4C–4C”). Unexpectedly, *Nrg1III*^*tg*^//*Lama*2^−/−^ sciatic nerves had more severe radial sorting defects than *Lama*2^−/−^ mice (Fig 4A), with a 3-fold increase in the number of unsorted axon bundles per sciatic nerve cross section (Fig 4C). This difference was present already at early stages in P5 sciatic nerves (S1A and S1B Fig). Thus, Nrg1III overexpression enhances the *Lama*2^−/−^ phenotype. Phenotypic enhancement suggests that the overexpressed gene, in our case Nrg1III, stimulates a pathway that is inhibited by the loss-of-function gene, in our case *Lama*2 [37,38]. Thus, a possible explanation is that Lm211 limits Nrg1III signaling pathways, thereby preventing precocious myelination of small-diameter fibers prior to the completion of axonal sorting and formation of an appropriate 1:1 relationship. In agreement with this hypothesis, unsorted bundles in sciatic nerves of *Nrg1III*^*tg*^//*Lama*2^−/−^ mice often contained axons that were myelinated by SCs before reaching the promyelinating 1:1 stage (Fig 4D, arrows).

Perturbed SC number can cause radial sorting defects [39]. Since both Lm211 and Nrg1III influence SC proliferation and survival [16,40], we asked if these parameters were synergistically altered in the double mutants and could explain the severe radial sorting phenotype. During radial sorting at P5, double mutants showed no increase in the percentage of TUNEL-positive nuclei or statistically significant decrease in phosphorylated-histone3 (P-H3) positive nuclei, and cell density was not changed (S1C Fig). At P16, there was a trend for increased apoptosis and decreased proliferation in the double mutants, but the changes were minimal (less than 0.4% apoptotic cells) and did not reach statistical significance. At P16, we also detected a decrease in cell density in *Nrg1III*^*tg*^ mice, probably due to thicker myelin, and an increase in cell density in *Nrg1III*^*tg*^/*Lama*2^−/−^, probably due to the reduction of myelinated fibers (S1D Fig). Overall, differences in SC number do not appear to be a major cause for the increased radial sorting defects observed in *Nrg1III*^*tg*^/*Lama*2^−/−^ mice.

### The regulation of myelin thickness by Lm211 in Nrg1III-overexpressing mice depends on axon caliber

**Fig 5:**
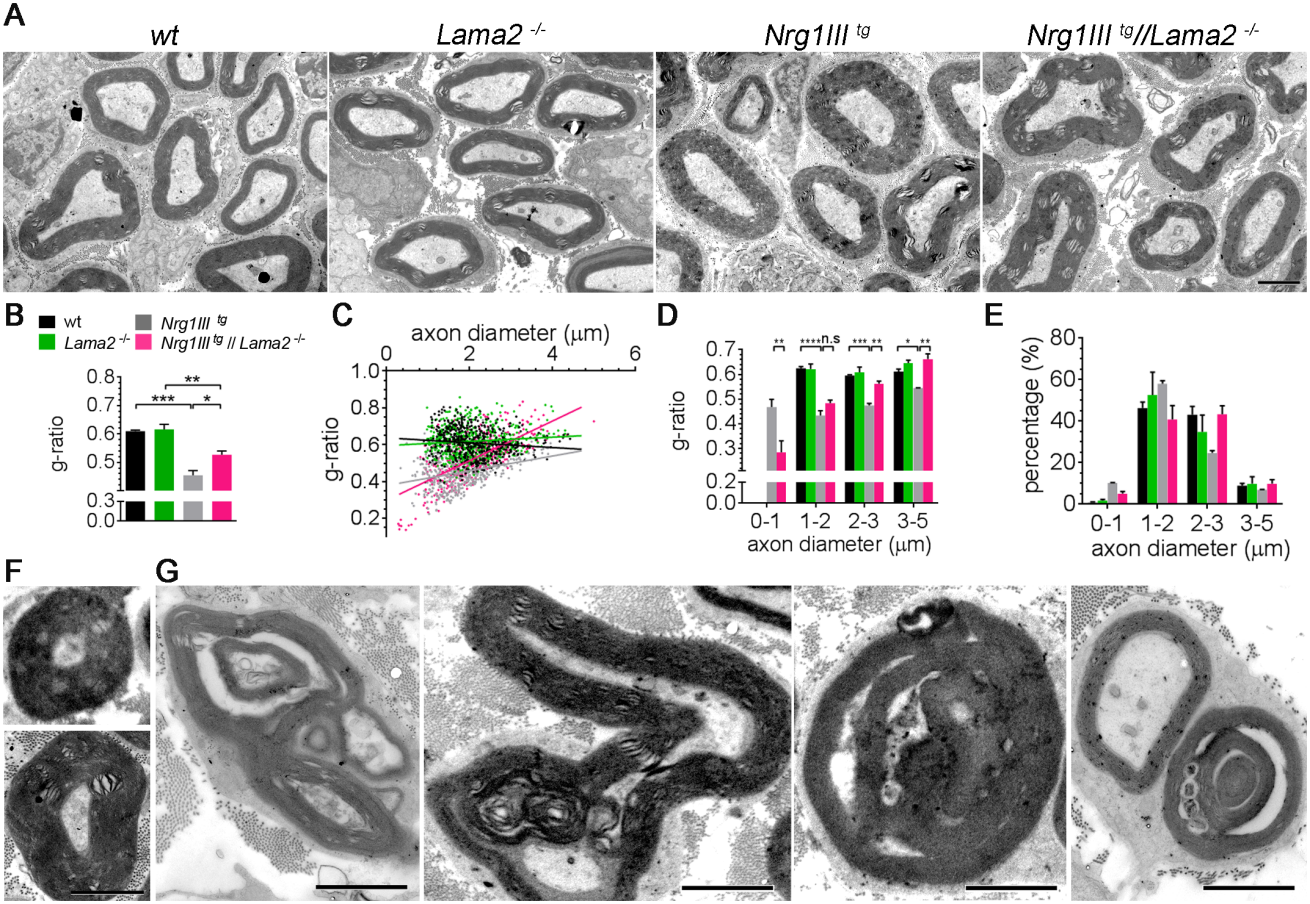
Laminin α2β1γ1 (Lm211) modulates myelin thickness in *Nrg1III*^*tg*^ sciatic nerves. (A) Postnatal day 16 (P16) electron micrographs of sciatic nerves of the indicated genotypes. (B) G-ratios were calculated from at least 150 myelinated axons per mouse (*n* = 3). *Nrg1III*^*tg*^ mice show decreased g-ratio, and *Nrg1III*^*tg*^//*Lama*2^−/−^ mice show an intermediate g-ratio between wild-type and *Nrg1III*^*tg*^. Scatter plot display (C) or distribution (D) g-ratios of individual fibers as a function of their axon diameter. The g-ratio of double mutants is opposite between small and large fibers, and the increase in myelin thickness is mainly observed in small axons. (E) Distribution of the diameter of myelinated axons in the indicated genotypes at P16. (F, G) Examples of aberrantly myelinated axons in *Nrg1III*^*tg*^//*Lama*2^−/−^ mice. In F, axons smaller than 1 μm are surrounded by a thick myelin sheath. In G, examples of redundant myelin are shown. Bar = 2 μm in A, F, G. The numerical data used in B–E are included in S1 Data.

Despite the severe radial sorting defects described in *Nrg1III*^*tg*^//*Lama*2^−/−^ nerves, some axons were myelinated, giving us the opportunity to measure myelin thickness. As before, *Lama2*^−/−^ nerves at P16 had normal g-ratios, and, as reported, *Nrg1III*^*tg*^ sciatic nerves had decreased g-ratios due to increased myelin thickness (Fig 5A and 5B). In the double *Nrg1III^tg^*//*Lama2*^−/−^ nerves, the overall average g-ratio was intermediate between wild-type and *Nrg1III^tg^* (Fig 5A and 5B), but plotting the g-ratio as function of the axon diameter revealed that removal of Lm211 further decreased the g-ratio of small fibers while progressively restoring to normal values the g-ratio of larger fibers (Fig 5C and 5D). This indicates that, as seen in *Nrg1III*^+/−^ mice (Fig 3), inhibition of Nrg1-induced myelination by Lm211 is predominant in small fibers. Notably, axons much smaller than 1 μm, which should not be myelinated, were often surrounded by a thick myelin sheath in double mutant nerves (Fig 5F). In *Nrg1III*^*tg*^//*Lama*2^−/−^ nerves, even large fibers with normal or thin myelin sheaths often displayed abnormal and redundant myelin, with infolding and signs of myelin degeneration (Fig 5G). These dysmyelinating features were also occasionally observed in *Nrg1III*^*tg*^ animals and are characteristic of certain forms of hereditary neuropathies, including those associated with deficiency of Lm211 [26]. Taken together, these data further substantiate the notion that Lm211 inhibits Nrg1III to prevent inappropriate myelination of small, unmyelinated axons and to limit myelin thickness in small caliber axons and the formation of redundant myelin in general.

### Loss of Lm211 increases PKA activation

The phenotypic enhancement shown by the genetic experiments described above suggests that Lm211 inhibits a pathway or a substrate that is normally stimulated by Nrg1III to promote myelination. In an attempt to find the pathway or substrate that it is inhibited by Lm211, we first reevaluated published work and performed experimental analysis that showed that Lm211 does not inhibit ERK or Akt (S2 Fig).

**Fig 6:**
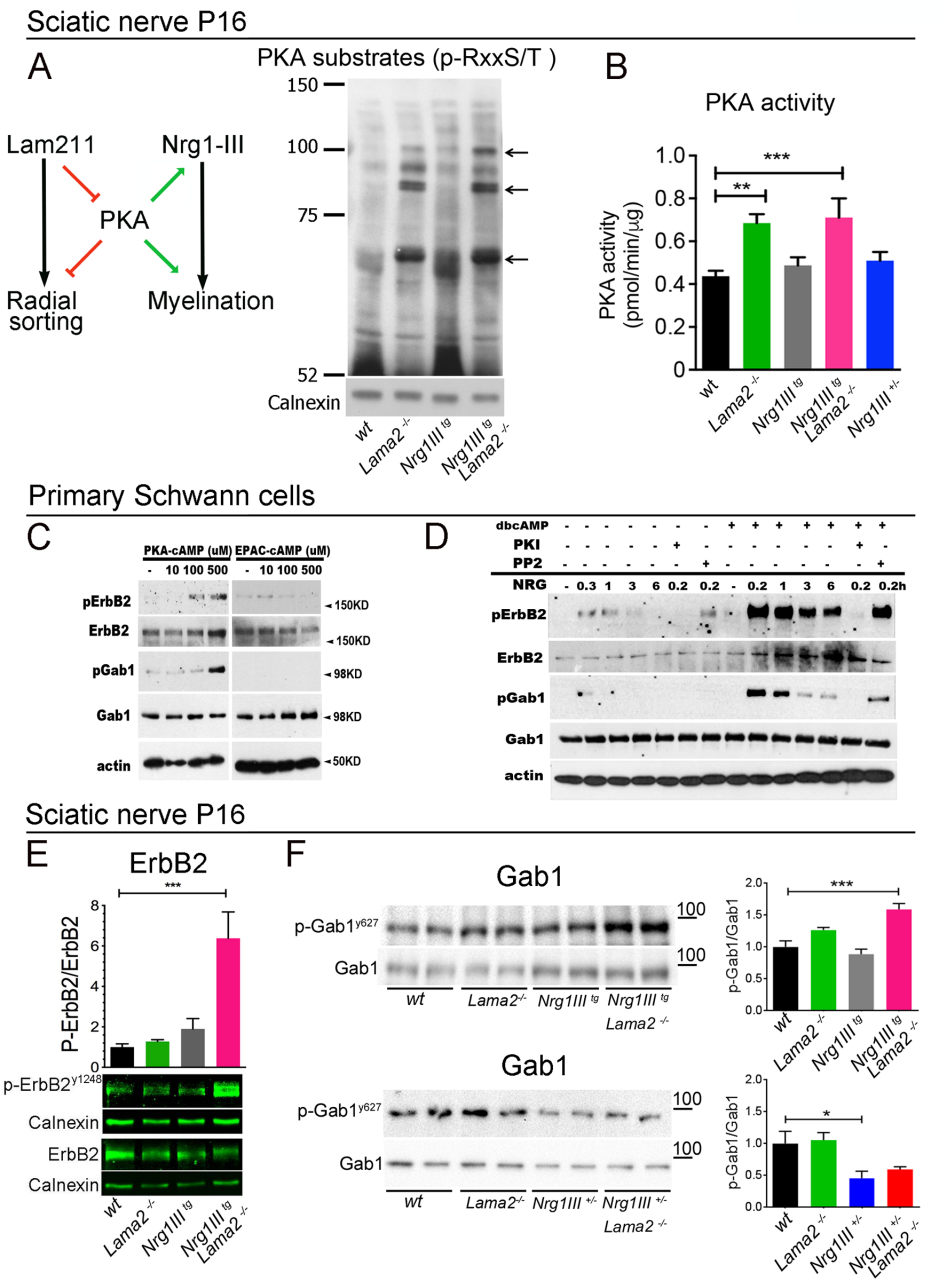
Increased protein kinase A (PKA), ErbB2, and Grb2-Associated Binder-1 (Gab1) activation in *Nrg1III*^*tg*^*//Lama2*^*−/−*^ sciatic nerves. (A) Schematic representation of the hypothesis that laminin α2β1γ1 (Lm211) inhibits neuregulin 1 type III (Nrg1III) promyelin signaling by negatively regulating PKA. Western blot of PKA phospho-substrates in P16 sciatic nerves. The image is representative of 3 experiments. (B) Measurement of PKA activity in sciatic nerves at P16 from the indicated genotypes. PKA is more active in *Lama*2^−/−^ and *Nrg1III*^*tg*^//*Lama*2^−/−^ nerves (*n* = 3 or more, **p < 0.01, ***p < 0.001 by 1-way ANOVA with Bonferroni multiple comparison test). (C) Treatment with a PKA-selective agonist (6-Bnz-cAMP), but not exchange protein directly activated by cAMP (EPAC) agonist (8-pCPT-2-O-Me-cAMP) for 3 days increases the levels of pErbB2 and pGab1 without Nrg1 treatment in primary SCs. The image is representative of 3 experiments. (D) Representative western blots showing the sensitization of the ErbB2-Gab1 pathway in response to Nrg1 following dbcAMP. Primary Schwann cells (SCs) in the presence (right 7 lanes) or absence (left 7 lanes) of dbcAMP for 3 days were exposed to Nrg1 (50 ng/ml) for the indicated time (h, hour). Where indicated, PKI166 (1 μM) or PP2 (1 μM) was used to pretreat cells before Nrg1 stimulation. Phosphorylation of ErbB2 and Gab1 was significantly enhanced following dbcAMP treatment and suppressed after PKI166 treatment. (E-F) Western blot analysis of ErbB2 (E) or Gab1 (F) phosphorylation in sciatic nerves of the indicated genotypes at P16. ErbB2 and Gab1 phosphorylation are increased only in *Nrg1III*^*tg*^//*Lama*2^−/−^ sciatic nerve. The experiments were repeated at least 3 times on 6 animals per genotype (E) or 3 times on 2 to 6 different animals per genotype (F). *p < 0.05, ***p < 0.001 by 1-way ANOVA with Bonferroni multiple comparison test. The numerical data used in B, E-F are included in S1 Data.

We next turned to PKA, which is required in parallel to Nrg1III to achieve full Egr2 activation and myelination [4–6]. PKA is a good candidate molecule because its hyperactivity causes a phenotype similar to that observed in *Nrg1III*^*tg*^/*Lama*2^−/−^ mice: an arrest in radial sorting with some promyelinating SCs undergoing premature myelination [41]. In addition, PKA may be activated by Nrg1 [42,43] and by Gpr126, a g-coupled protein receptor that binds various ligands, including Lm211[44,45]. We hypothesized that PKA, or one of its substrates, may be normally inhibited by Lm211 and that the phenotype of *Nrg1III*^*tg*^/*Lama*2^−/−^ mice may be due to excessive Nrg1III-driven promyelinating signals, plus disinhibited PKA signaling (Fig 6A).

To test this idea, we first evaluated the amount of substrates phosphorylated by PKA in sciatic nerves using an antibody that recognizes the PKA-phosphorylated consensus motif RxxS/T (p-Sub antibody). This revealed a discrete number of bands, many of which were upregulated in nerves deficient in Lm211 but not in those with Nrg1III overexpression (Fig 6A). We also measured PKA activity directly in sciatic nerves at P5 and P16 and confirmed that PKA was hyperactive in the absence of Lm211 at both timepoints (Fig 6B and S2 Fig). In contrast PKA activity was normal in *Nrg1III*^*tg*^ at P16, higher at P5, and normal in *Nrg1III*^+/−^ nerves, suggesting that Nrg1III may not regulate PKA in SCs in vivo. PKA is regulated by levels of cAMP or by lipids and peptides in a cAMP-independent fashion [46–49]. To determine if the hyperactivity of PKA in Lm211 null SCs was caused by an increase in cAMP, we measured cAMP concentration in sciatic nerves of mutant mice. Interestingly, the levels of cAMP at P16 and P5 were low in *Lama*2^−/−^ nerves and normal in *Nrg1III*^*tg*^ (S2 Fig). Overall, these results indicate that Lm211 inhibits PKA activation, possibly by a cAMP-independent mechanism.

### Nrg1III overexpression in SCs results in excessive activation of the ErbB2-Gab1 promyelinating pathway only when combined with Lm211 deficiency

We next investigated which steps of the Nrg1III signaling cascade were influenced by the Lm211 and PKA axis. In vitro, PKA phosphorylates ErbB2 [50], thus, one PKA substrate that accumulates in *Lama*2^−/−^ nerves could be the Nrg1 receptor ErbB2/ErbB3 itself. Interestingly, sustained treatment of cultured SCs with a PKA-selective agonist increased phospho-ErbB2 in a dose-dependent manner, in the absence of Nrg1 in the culture media (Fig 6C). In contrast, an agonist of exchange protein directly activated by cAMP (EPAC) did not increase phospho-ErbB2. Higher doses and longer treatment were required to activate ErbB2, similar to the conditions required to promote SC differentiation and *Egr2* expression [5,51,52], while short treatments did not activate ErbB2, as previously reported [50]. The PKA-selective agonist also induced phosphorylation of Grb2-Associated Binder-1 (Gab1), an adaptor protein that is phosphorylated upon Nrg1III/ErbB signaling in SCs and is required for myelination [53](Fig 6C). These data suggest that PKA can directly transactivate the ErbB2-Gab1 axis independently of Nrg1III. It is known that cAMP-PKA is also required to amplify Nrg1 signals in SCs [4–6]. To confirm this, we exposed primary SCs to either Nrg1 alone or Nrg1 and dbcAMP and showed that the phosphorylation of ErbB2 and Gab1 were enhanced if the SCs were exposed to both Nrg1 and dbcAMP (Fig 6D). To confirm that Gab1 phosphorylation was downstream of Nrg1-ErbB signaling, we pretreated SCs with the ErbB2 inhibitor PKI166 [54] and showed that Gab1 phosphorylation was inhibited. In contrast, treatment with the Src-kinase inhibitor PP2 did not have any effect (Fig 6D). Thus, PKA can directly activate ErbB2, and cAMP sensitizes the response of SCs to the Nrg1III-ErbB2-Gab1 pathway, at least in vitro. To ask if ErbB2 and Gab1 were modulated by Lm211 in vivo, we next measured their phosphorylation status in mutant sciatic nerves. Strikingly, phosphorylation of ErbB2 and Gab1 was not increased in nerves of *Nrg1III*^*tg*^, probably due to the presence of an intact Lm211 “brake;” however, deleting Lm211 in the context of Nrg1III overexpression (*Nrg1III*^*tg*^/*Lama*2^−/−^) significantly increased ErbB2 and Gab1 phosphorylation (Fig 6E and 6F). A similar trend was observed at P5 (S4 Fig). Overall, these data support the view that Lm211, via inhibition of PKA, reduces the output of Nrg1 signaling in SCs in vivo.

### PKA activation in Lm211-deficient SCs is associated with increased expression of Oct6 and Egr2

**Fig 7:**
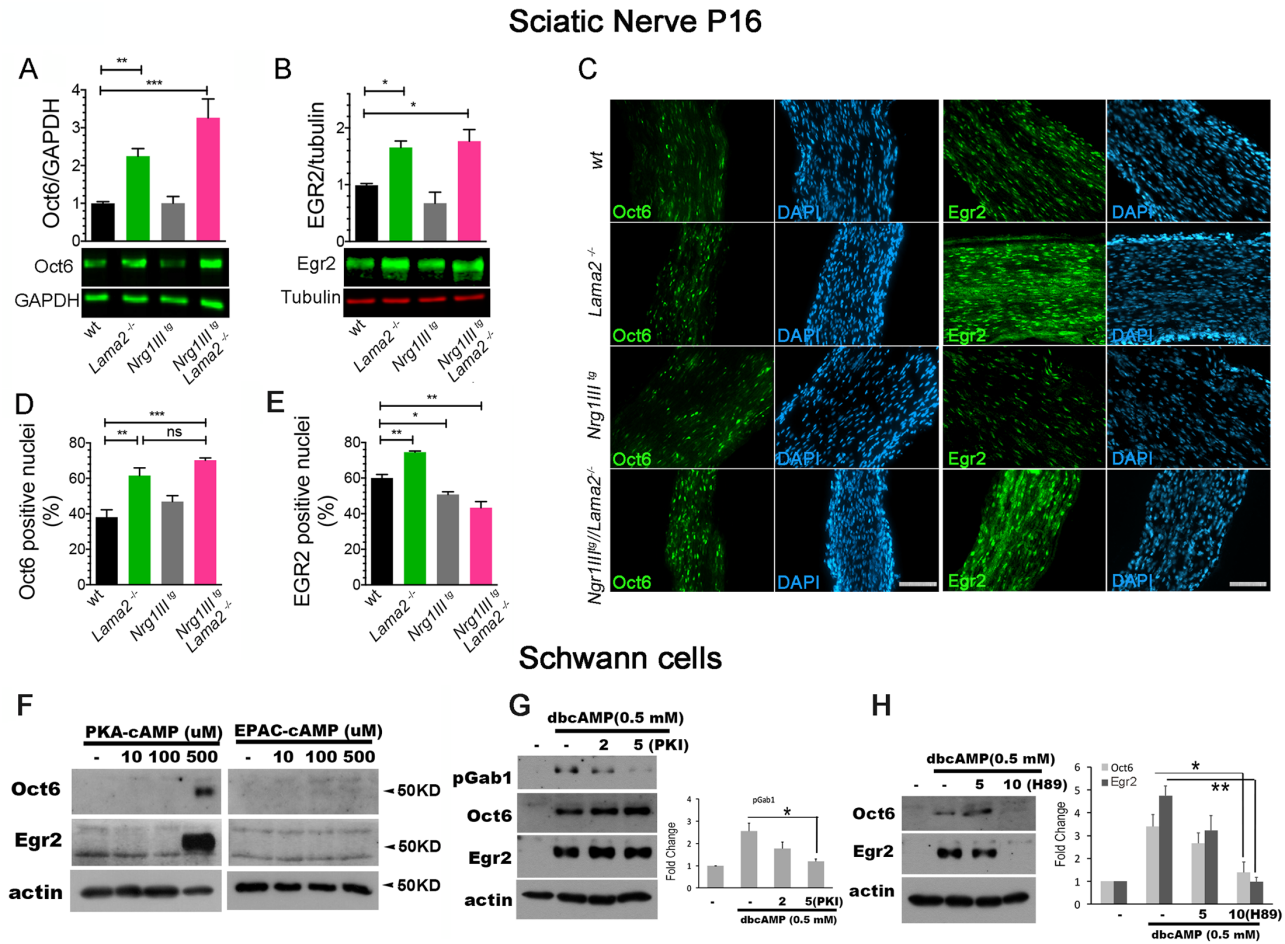
Oct6 and Egr2 expression are increased in *Lama*2^−/−^ and *Nrg1III*^*tg*^//*Lama*2^−/−^ mutants. (A, B) Western blot from postnatal day 16 (P16) sciatic nerves shows that the levels of Oct6 and Egr2 are increased in *Lama*2^−/−^ and *Nrg1III*^*tg*^//*Lama*2^−/−^ nerves but not when neuregulin 1 type III (Nrg1III) is overexpressed alone. The experiments were repeated at least 3 times on 5 (Oct6) or 4 (Egr2) different animals per genotype. *p < 0.05; **p = 0.006; ***p < 0.0001 by 1-way ANOVA with Bonferroni multiple comparison test. (C) Sciatic nerve longitudinal sections at P16 from the indicated genotypes were stained for Oct6 or Egr2 (green) and DAPI (blue). (D, E) Quantification of the fraction of positive Oct6 and EGR2 nuclei. Three animals per genotype were analyzed. *p < 0.05; **p < 0.005; ***p = 0.0007. A, B, D, E statistic by 1-way ANOVA with Bonferroni posthoc test for individual comparisons. (F) Western blot analysis showing dose-dependent induction of Egr2 and Oct6 by protein kinase A (PKA)-selective agonists in primary Schwann cells (SCs) in culture. (G) The inhibition of ErbB2 with PKI166 suppresses 3′-5′-cyclic adenosine monophosphate (cAMP)-induced Grb2-Associated Binder-1 (Gab1) phosphorylation, but not the expression of Oct6/Egr2 in primary SCs. (*n* = 3, *, *p* < 0.05 by Student *t* test. (H) PKA inhibition with H89 suppresses cAMP-induced expression of Oct6/Egr2 in primary SCs. (*n* = 3, *, *p* < 0.05, **, *p* < 0.01 by Student *t* test). Bar = 100 μm in C. The numerical data used in A-B, D-E, G-H are included in S1 Data.

We next tested if the Oct6 and Egr2 transcription factors, downstream of PKA and Nrg1, were modulated in our system. By western blot (WB), the levels of both Oct6 and Egr2 were increased in *Lama2*^−/−^ and double mutant nerves at P16 (Fig 7A and 7B), but not in *Nrg1III*^*tg*^ nerves, suggesting as before that Lm211 inhibition has to be released to drive excessive Nrg1III-induced SC differentiation. A similar trend was observed for Egr2 in double mutants earlier in development, corroborating that SCs may initiate premature differentiation (S4 Fig). The number of Oct6 and Egr2 positive nuclei were also increased in Lm211-deficient nerves (Fig 7C–7E), but the number of Egr2 positive nuclei was decreased in double mutants, likely due to the arrested development with a reduced number of SCs reaching the promyelinating stage (see Fig 4). Both cAMP and Nrg1 are required to induce sustained expression of Egr2 in SCs in culture [5,6,55], and cAMP-PKA increases ErbB2 phosphorylation both in response to Nrg1 [50] and independently of Nrg1 (Fig 6C). Therefore, we hypothesized that PKA could also induce Oct6 and Egr2 expression independently of Nrg1. To test this, we asked if treatment of SCs with a PKA agonist induced Oct6 and Egr2 protein levels. Indeed, exposure of SCs to the specific PKA agonist induced both transcription factors (Fig 7F). This induction was present in the absence of Nrg1, and was not seen with an EPAC-specific agonist. To test if ErbB2 phosphorylation was required for the induction of Oct6 and Egr2, we treated rat SCs with dbcAMP and analyzed Oct6/Egr2 expression after inhibition of ErbB2 with a specific inhibitor, PKI166. ErbB2 inhibition caused the expected block in dbcAMP-induced Gab1 phosphorylation, but did not alter Oct6 or Egr2 levels (Fig 7G). In contrast, PKA inhibition with H89 suppressed the induction of both transcription factors by dbcAMP (Fig 7H). These results suggest that in cultured SCs, PKA may activate Oct6 and Egr2 independently of Nrg1.

### Increased PKA activation amplifies promyelin signals downstream of Nrg1III

**Fig 8:**
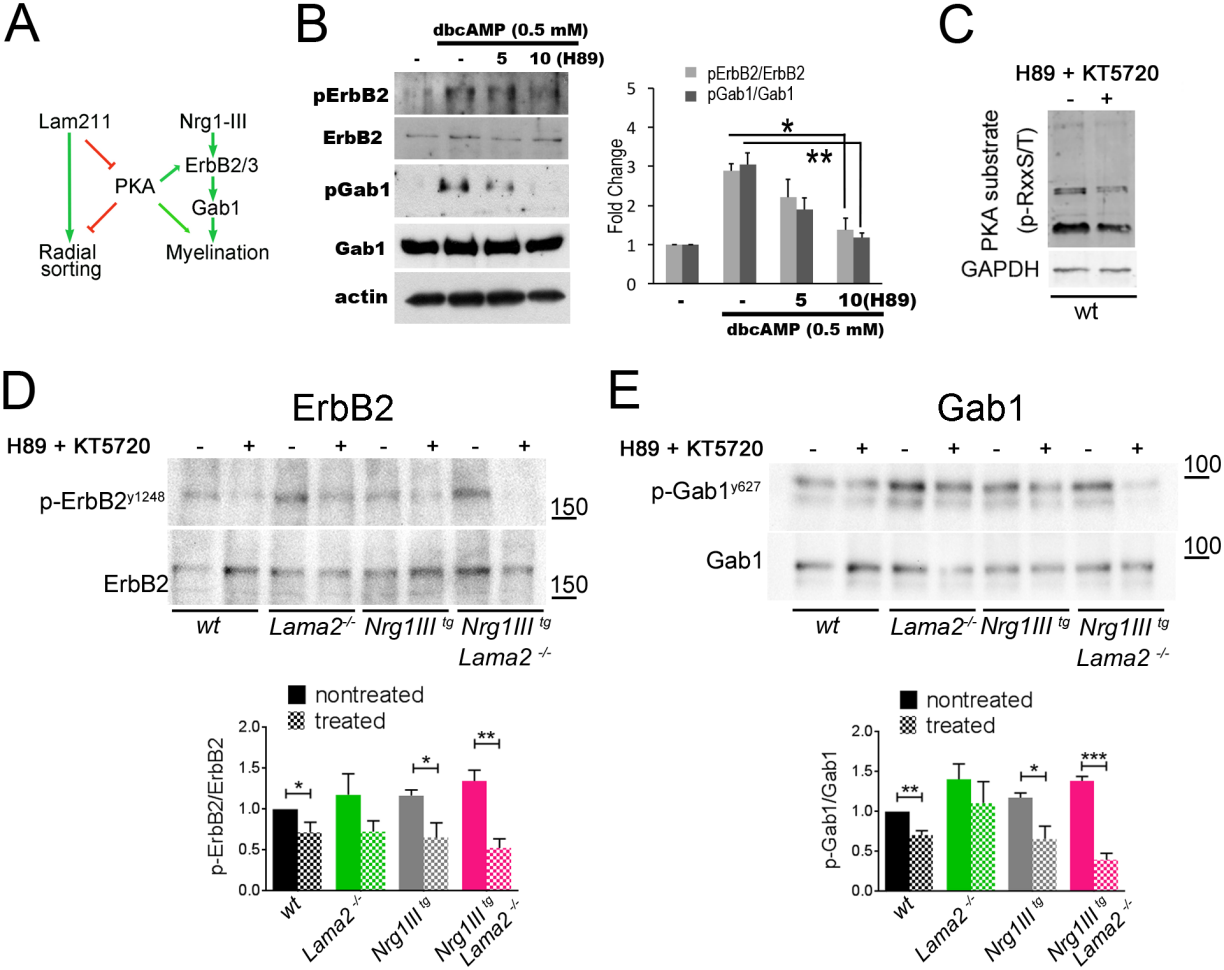
Laminin α2β1γ1 (Lm211) inhibits neuregulin 1 type III (Nrg1III)-induced ErbB2/3 and Grb2-Associated Binder-1 (Gab1) activation by negatively regulating protein kinase A (PKA). (A) Schematic representation of the hypothesis that Lm211 inhibits ErbB2/3 and Gab1 downstream of Nrg1III by negatively regulating PKA. (B) The PKA inhibitor H89 dose-dependently suppressed dbcAMP-induced ErbB2 and Gab1 phosphorylation in rat Schwann cells (SCs). dbcAMP was used for 3 days (*n* = 3, *p < 0.05; **p < 0.001 by Student *t* test). (C) Intermuscular injection of HB9 and KT5720 for 4 days reduced PKA activity, revealed by a decrease in PKA phospho-substrates. (D, E) Similar treatment significantly reduced ErbB2 and Gab1 activation in *Nrg1III*^*tg*^//*Lama*2^−/−^ and, to a lesser extent, in the other genotypes. The experiments were repeated at least 5 (D) or 3 (E) animals per genotype. *p < 0.05, ***p < 0.005, ***p < 0.001 by Student *t* test. The numerical data used in B, D-E, are included in S1 Data.

We showed that in *Nrg1III*^*tg*^//*Lama*2^−/−^nerves, ErbB2 and Gab1 are more active, and this is associated with an increase in PKA activation due to Lm211-deficiency. To determine if the effect of Lm211 loss is indeed mediated by PKA activation (Fig 8A), we inhibited PKA activity in vitro and in vivo using the selective PKA antagonists H89 and KT5720 and asked if this was sufficient to decrease ErbB2 and Gab1 phosphorylation. In cultured SCs, H89, dose-dependently suppressed the activation of ErbB2 and Gab1 (Fig 8B). In vivo, we injected H89 and KT5720 beneath the gluteus superficialis and biceps femoris muscles, in which the sciatic nerve resides, every day from P3 to P6 and sampled the nerves at P7. The contralateral side was injected with DMSO and used as control. This procedure has been shown to effectively deliver pharmacological treatment within sciatic nerves [56] and indeed we could observe a reduction of PKA substrate phosphorylation in nerves treated with the inhibitors (Fig 8C). Strikingly, the inhibitors significantly decreased ErbB2 and Gab1 activation in *Nrg1III*^*tg*^//*Lama*2^−/−^ mice, indicating that PKA directly contributes to the promyelinating signals initiated by Nrg1III in Schwann cells (Fig 8D and 8E). The expression of Oct6 and Egr2 instead could not be consistently modulated by this short pharmacological treatment (S5 Fig). Taken together, our results strongly suggest that Lm211, through inhibition of PKA, limits the activation of promyelinating signaling molecules such as ErbB2 and Gab1 in SCs.

**Fig 9:**
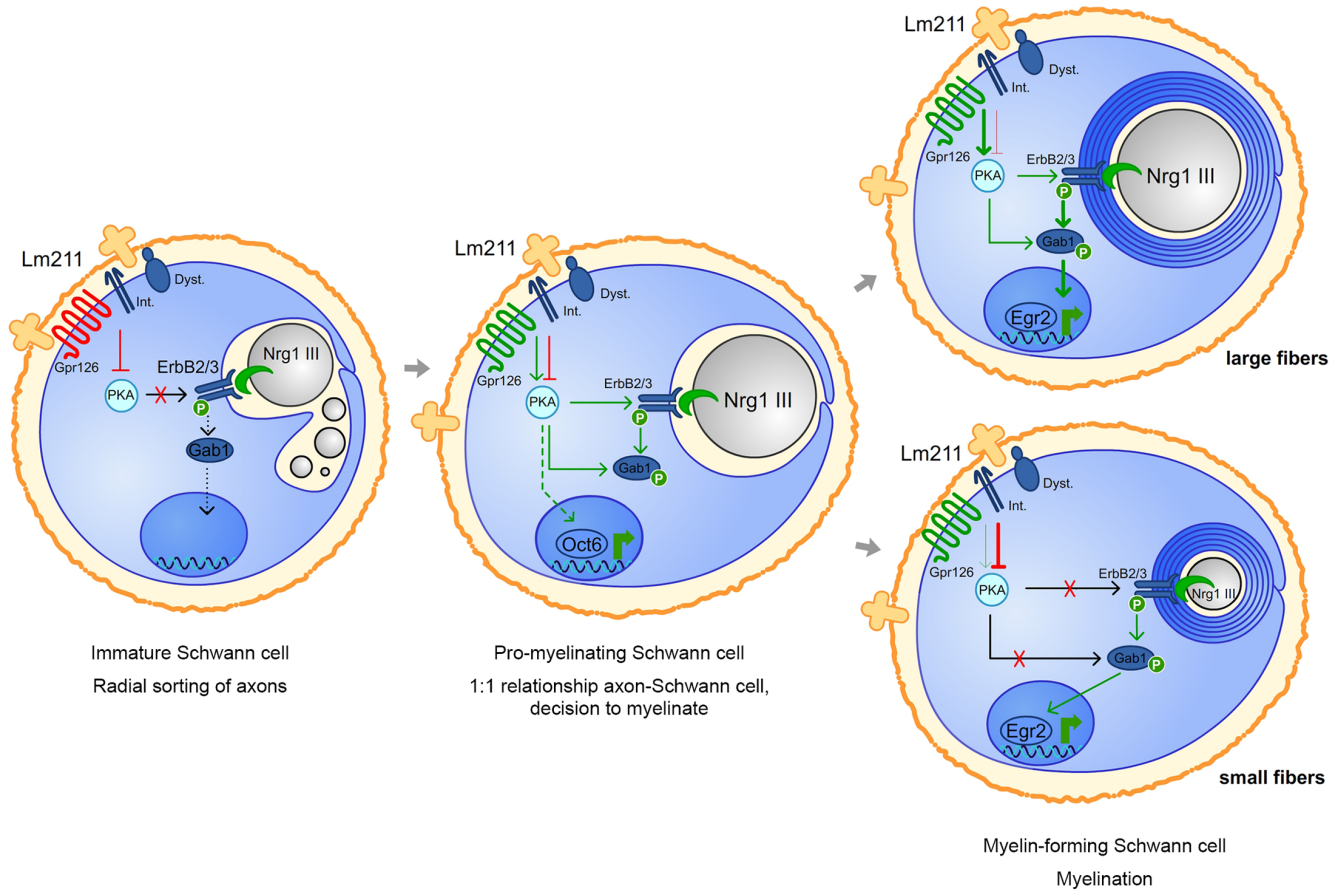
Model depicting how laminin α2β1γ1 (Lm211) and neuregulin 1 type III (Nrg1III) signaling are integrated during SC development. In immature SCs, Lm211, via one or more of its basal lamina receptors (Int = integrins, Dystroglycan = Dyst, Gpr126), inhibits protein kinase A (PKA) and prevents Nrg1III from triggering myelination during radial sorting. In promyelinating cells, after radial sorting is finished and the 1:1 relationship with axons larger than 1 μm has been achieved, PKA is activated by Gpr126 independently of Lm211 [45,57] and contributes to Nrg1III signaling and to the expression of Oct6. In large myelinating fibers (above), Lm211 inhibition is overcome, PKA is fully active, and cooperates with Nrg1III to activate ErbB2, Grb2-Associated Binder-1 (Gab1), and Egr2. In small myelinated fibers (bottom), Lm211 inhibition of PKA persists and prevents excessive Nrg1III-driven myelination.

Overall, based on our data, we conclude that Lm211 inhibits Nrg1III via PKA in several instances: during radial sorting, to prevent premature SC differentiation; at the onset of myelination, to prevent myelination of fibers smaller than 1 μm; and during myelination, to limit myelin thickness in small fibers (Fig 9).

## Discussion

Our data clarify for the first time that Lm211 modulates and can even inhibit, rather than promote, myelination in vivo. SC development depends on a discrete number of extrinsic signals originating from the axon and the ECM [58,59]. How SCs coordinate these different signals to achieve myelination is poorly understood. Here, we focused on 2 of the major extrinsic signals: the axonal molecule Nrg1III and the ECM component Lm211. Although these molecules have been known for years to be important for SC development, it was not known how these signals on 2 opposite surfaces of the SC collaborate to achieve myelination. We show that Lm211 has an inhibitory role on several downstream effectors of the Nrg1 pathway. We show that this effect is mediated by inhibition of PKA, that PKA is hyperactive in the absence of Lm211, and that this leads to overactivation of ErbB2 and Gab1 when combined with Nrg1III overexpression, resulting in an arrest of radial sorting and in premature myelination. Taken together, our results strongly indicate that Lm211 limits PKA activation and blocks this parallel pathway that needs to converge with Nrg1III to initiate myelination. Our data begin to clarify how Nrg1III can regulate such different SC responses: proliferation, survival, and myelination, using the single receptor ErbB2/ErbB3. We propose that deposition of Lm211 in the basal lamina modulates the SC response to axonal Nrg1III to favor proliferation, survival, and axonal ensheathment during radial sorting while inhibiting myelination, effectively modulating the Nrg1 response during development from a proliferative to a myelinating signal.

Based on our data, Lm211 limits the response of SC to Nrg1III at multiple steps of development. In immature SCs, Lm211 promotes radial sorting and inhibits Nrg1-driven premature myelination. In promyelinating SCs, Lm211 prevents Nrg1-driven inappropriate myelination of axons smaller than 1 μm and limits myelin thickness on small axons (Fig 9). Thus, although Lm211 function predominates during radial sorting and Nrg1 predominates during myelination, both are required to fine-tune the onset and extent of each developmental step. During radial sorting, the levels of Nrg1III on axons are probably read by ErbB2/3 receptors as a binary fate choice (myelination versus nonmyelination): above a threshold of Nrg1III SCs start myelination, as increasing the expression of Nrg1III can switch the fate of nonmyelinated axons to myelinated [27]. However, our data indicate that Lm211 increases the Nrg1III threshold required for myelination. In contrast, expressing a dominant-negative form of integrin β1 in oligodendrocytes increases the threshold for myelination, but it is unclear if this is mediated by the Lm211 ligand and if this function is linked to a modulation of Nrg1III signaling also in the central nervous system [60].

As summarized above, we provide evidence that Lm211 and Nrg1III have reciprocal roles during radial sorting and myelination. This could suggest that there is a reciprocal inhibition also from Nrg1III to Lm211, and that this inhibition may be required to terminate radial sorting driven by laminin. Nrg1III could conceivably suppress the sorting behavior of SCs also via PKA (dotted-arrow in S3B Fig). This could explain the drastic impairment in radial sorting observed in *Nrg1III*^*tg*^//*Lama*2^−/−^ mice, and there is evidence in the literature indicating that Nrg1 activates PKA [42,43]. However, our results in vivo were conflicting, with Nrg1III overexpression increasing PKA activity at P5 but not at P16, and Nrg1III haploinsufficency not reducing PKA activation. Thus, our data did not allow us to conclusively confirm this idea.

Our data indicate that myelin thickness is also modulated by Lm211, but interestingly, the effect is opposite in small- and large-caliber fibers. This could be potentially be explained by the fact that Lm211 uses different receptors (α6β1 and α6β4 integrin, dystroglycan, Gpr126) that may exert positive and negative effects, which when removed together by virtue of removing the common Lm211 ligand, do not affect myelin thickness. It is also interesting to note that the axonal sorting defects in Lm211 mutants are more severe in motor than sensory roots, suggesting a higher dependence of motor fibers on Lm211. It follows that it will be interesting to explore if the different effects of Lm211 on myelin thickness of large and small fibers may coincide with a different effect of laminin–Nrg1III interactions on motor versus sensory nerve fibers. Finally, the regulation of myelin thickness by Nrg1III is also more evident in small fibers [28]. Up to now, the role of Lm211 in the control of myelin thickness was controversial, and this can be explained by the fact that Lm211 has different effects in small and large fibers and that the modulating role of Lm211 cannot be revealed when the Nrg1III axis is intact.

Why were laminins considered only as a promoter of myelination throughout the years? Multiple experiments were interpreted to show that laminin is necessary for SC to myelinate in vitro because formation of myelin was used as an endpoint [20,21,32], rather than considering radial sorting and myelination as 2 distinctive steps in development. Indeed, when radial sorting was examined by EM in these studies, SCs were blocked at the immature and not at the promyelinating stage. Similarly, in vivo, it was reported that ErbB2 phosphorylation was decreased in the nerves of mice lacking all laminins in SCs [35]. SCs in these mutant mice are arrested at the immature stage, and they are more undifferentiated than their wild-type counterparts, likely explaining why ErbB2 phosphorylation appeared to be decreased. Finally, it was previously reported that loss of Lm211 leads to a reduction in myelin thickness in the same *Lama2*^−/−^ animal model that we used [23]. This discrepancy can be explained by the fact that myelin thickness, rather than g-ratio was measured, using an automated program and light microscopy. In our hands, only the analysis of measurement of g-ratio using EM could reliably and consisistently reveal the changes in g-ratio in small caliber fibres. Also, in previous studies, myelin thickness was not evaluated as a function of axonal diameter, potentially confusing the results, based on the differences that we have observed between small- and large-caliber fibers. This, together with the fact that normal myelin thickness was also reported in other Lm211 mutants generated in the past [15,16,61], make us confident of our conclusion that Lm211 deletion alone is not sufficient to influence myelin thickness.

The molecular mechanisms through which Lm211 inhibits PKA and PKA promotes myelination are only partially understood. Lm211 binds Gpr126, a G protein-coupled receptor that increases cAMP signaling and is required for peripheral myelination [44,45]. One of the downstream effectors of GPR126-cAMP is PKA, and together they are required to activate Egr2 expression and initiate myelination in a Nrg1III-dependent manner [4]. We originally postulated that Lm211 decreases PKA activity by regulating Gpr126 and cAMP. Gpr126 binds Lm211 to regulate the release of an inhibitory fragment with context-dependent effects on the levels of cAMP[45]. However, cAMP levels were decreased in mutants lacking Lm211, suggesting that the net effect of Lm211 on Gpr126 is stimulatory for cAMP production. Lm211 may indirectly favor binding of Gpr126 to the activating ligands collagen IV [57] and cellular prion protein on axons [62]. Collagen IV binding may depend on Lm211 because laminins favor basal lamina polymerization [63,64], and prion binding may depend on Lm211 for proper radial sorting and contact between SCs and axons [15]. On the other hand, our finding that cAMP was low in Lm211-deficient nerves also suggests that the increased activation of PKA is cAMP-independent. There are several examples of cAMP-independent PKA activation in other cell types [46–49], and Lm211 receptors such as α6β4 integrins and dystroglycan could potentially be involved [24,65]. The mechanism by which PKA regulates myelination has been the subject of recent work, and it is only partially clarified. PKA in SCs regulates the cytoskeleton [66], signaling molecules, and transcription factors, such as members of the CREB family and Egr2 [4,5]. How PKA induces Egr2 is unclear. One possibility is by activating NfkB and inducing Oct6. A cytoplasmic pool of PKA phosphorylates the p65 NfkB subunit on Serine 276 in SC [7], and, interestingly, this regulation may be cAMP-independent [67,68]. NF-kb is required for axonal ensheathment and activation of Oct6 and binds the chromatin remodeler Brg1, which is essential for myelination [69,70]. Oct6, in turn, activates Egr2 expression [71,72]. Therefore, it is tempting to speculate that Gpr126, cAMP, PKA, NfkB, and Oct6 are all part of a transient, linear pathway that switch-on Nrg1III-driven myelination, and that Lm211, collagen IV, and prion proteins modulate it.

Our data have implications for human diseases. Loss-of-function mutations in *LAMA*2 causes Congenital Muscular Dystrophy 1A, which include demyelinating peripheral neuropathies characterized by heterogeneous myelin thickness with focal hypermyelination, loss of nerve fibers, short internodes, and wide nodes of Ranvier [26,73]. While the mechanisms of the short internodes and wide nodes of Ranvier have been clarified [74–76], the molecular basis of the focal hypermyelination in these patients was unclear. Our finding that Lm211 inhibits Nrg1III signaling in small fibers could explain the effect of Lm211 deficiency in a neuropathic nerve, in which Nrg1 signals may be secondarily imbalanced. Similar alterations in the balance between Nrg1III and Lm211 signaling during myelination could explain other human neuropathies, such as Charcot-Marie-Tooth 4F and leprosy. The former is due to recessive mutations in periaxin, an interactor of the dystrophin-complex linked to dystroglycan in SCs. Charcot-Marie-Tooth 4F is also linked to hypermyelination and demyelination, possibly explained by the interrupted connection between dystroglycan and its ligand, Lm211, in the SC basal lamina. Similarly, the leprosy mycobacterium infects peripheral nerves by binding Lm211 in SCs and activates ErbB2 [77,78]. The resulting dedifferentiation and demyelination could be explained by concomitant hyperactivation of ErbB2 and inhibition of Lm211, which, as we have shown here, is deleterious for myelin-forming SCs.

## Materials and methods

### Transgenic mice

All experiments involving animals followed experimental protocols approved by the San Raffaele Scientific Institute Animal Care and Use Committee and Roswell Park Institute Animal Care and Use Committee. The approved protocols at San Raffaele (n. 363) and at the University of Buffalo/Roswell Park (UB1188M, UB1194M, UB1196R) adhered to the guidelines set forth by the “Guide For The Use of Laboratory Animals,” National Research Council, National Academy Press, Washington D.C., 1996. *Nrg1III*^+/−^ mice were characterized in [27] and were a gift from Drs. Talmage and Role at SUNY Stony Brook; *Nrg1III*^tg^ mice were characterized in [36]; *Lama2*^−/−^ mice were characterized in [23] and were a gift from Dr. Takeda, National Center of Neurology and Psychiatry, Tokyo. All animals used in this work were congenic into the C57/BL6N background. Genotyping of mutant mice was performed by PCR on tail genomic DNA, as described in [27,36]. For *Lama2*^−/−^, we used the following primers: 5′-CCCGTGATATTGCTGAAG-3′; 5′-CCTCTCCATTTTCTAAAG-3′; 5′-CAGGTGTTCCAGATTGCC-3′. PCR was carried out at 95°C for 45 s, 50°C for 45 s, followed by extension at 72°C for 60 s, for 30 cycles. The expected 246 nt product for the wild-type allele and 450 nt product for the mutant allele were separated on a 2% agarose gel.

### Morphological analysis

Mutant and control littermates were sacrificed at P5 and P16, and sciatic nerves were dissected. Semithin sections and EM analyses of sciatic nerves were performed as previously described [79]. The quantification of the number and the diameter of the axons in the unsorted bundles, the determination of g-ratios (axon diameter/fiber diameter) and the quantification of Remak bundles were performed on ultrathin sections. At least 3 animals per genotype were analyzed. Unsorted bundles were defined as groups of “naked” axons with no SC cytoplasm among them, and they contained some axons larger than 1 μM. All of the unsorted bundles in a section were counted. In contrast, Remak bundles differed from unsorted bundles because they contained ensheathed axons, all smaller than 1 μM. G-ratio were determined for at least 150 fibers chosen randomly. EM analyses on SC–DRG neurons cocultures from WT and CRD KO embryos following Lm211 treatment were performed as described [80].

### Cell cultures

Dorsal root ganglia (DRG) neurons were generated as described in [27]. Rat SCs (200,000 cells/coverslip) were added to established cocultures, and myelination was initiated by supplementing the media (Fetal Calf Serum [FCS] 10%, L-glutamine 2 mM, D-glucose 4 g/l, Nerve Growth Factor [NGF; Harlan, Bioproducts for Science] 50 ng/ml in MEM medium [Invitrogen]) with 50 μg/ml of recombinant Lm211 purified as described in [81,82] or obtained from (Biolamina) and dyalized. Primary SCs were isolated from the sciatic nerves of 4-day-old Sprague-Dawley pups according to [83]. To expand the SC population, cells were kept in growing medium: DMEM containing 1% FBS, Nrg1 30 ng/ml (human NRG1-β1 extracellular domain, R&D Minneapolis, MN), and forskolin 5 μM (Calbiochem, Gibbstown, NJ) for 2 to 4 generations. More than 95% of SC purity was verified based on their morphology and S100 immunoreactivity [54]. For experiments, cells were subcultured into 12 well dishes in growing medium, and after the cell density reached 70% confluency, they were kept for 3 days in differentiation medium: DMEM containing 1% FBS and dbcAMP (Dibutyryladenosine 3′,5′-cyclic monophosphate sodium salt, Bremen, Germany) without NRG1. PKA inhibitor H89 (Novartis, Basel, Switzerland) and ErbB2 inhibitor PKI166 (Novartis, Basel, Switzerland) were added after 24h of dbcAMP treatment and then left for 2 days in combination with dbcAMP (Figs 6 and 8). For the sensitization experiment (Fig 6), cells were first treated with cAMP for 3 days, then Nrg was added for the indicated time in the presence of cAMP. When indicated, PKI166 and PP2 (Calbiochem, Gibbstown, NJ), were added 30 min before Nrg1 treatment. 6-Bnz-cAMP and 8-pCPT-2-O-Me-cAMP were obtained from Biolog (Bremen, Germany). All other undesignated reagents were purchased from Sigma.

### Antibodies

All antibodies used were previously validated for the applications used. Antibodies against ErbB2 (sc-7301), pErbB2^Y1248^ (sc-12352-R) for western blot (WB) were from Santa Cruz Biotechnology. Anti-ErbB2 (4290, 1:1000 for WB), Gab1 (3232, 1:1000 for WB), p-Gab^Y627^ (3231, 1:1000 for WB), and Phospho-PKA Substrate (RRXS*/T*) (9621, 1:1000 for WB) were from Cell Signaling. Anti-Oct6 were either from Santa Cruz (sc-11661) or from (D. Meijer, University of Edinburgh, United Kingdom, 1:1000 for IHC and WB). Anti-Egr2 was either from Covance (PRB-236P) or from D. Meijer, University of Edinburgh, UK, 1:1000 for IHC and WB). p-Histone H3 (06–570, 1:200 for IHC) was from EMD Millipore. Anti-neurofilament M was from Covance (PKC-593P, 1:1000 for IHC) and Anti-MBP from V. Lee, University of Pennsylvania, USA (1:6 for IHC). Anti-calnexin (C4731, 1:4000 for WB), tubulin (T4026, 1:2000 for WB), GAPDH (G9545, 1:5000 for WB) and β-actin (A5441, 1:1000 for WB) were from Sigma-Aldrich. Secondary antibodies: goat antichicken DyLight 550, (Abcam, ab96948, 1:700 for IHC), goat antirabbit Alexa Fluor 488 (111-545-003, 1:700 for IHC) and goat antirabbit HRP (111-035-003, 1:5.000 for WB) goat antimouse HRP from Jackson ImmunoResearch; (SIGMA, A2554, 1:10000 for WB). Infrared secondary antibodies for quantitative WB analyses were obtained from LI-COR Biosciences and used 1:10,000 (goat antimouse IRDye 680 926–68070; goat antimouse IRDye 800 926–68070; goat antirabbit IRDye 680 926–68021; goat antirabbit IRDye 800 926–32211).

### Western Blot

Frozen sciatic nerves dissected from P5 and P16 mice were pulverized and resuspended in lysis buffer (95 mM NaCl, 25 mM Tris-HCl, pH 7.4, 10 mM EDTA, 10mM EGTA, 2% SDS, 1 mM Na3VO4, 1 mM NaF, 1% Protease Inhibitor Cocktail [Sigma-Aldrich]), 1% Phosphatase inhibitor cocktail C2 and C3 (Sigma-Aldrich), boiled for 5 min, and centrifuged 10 min at 17,000 g at 16°C. The protein concentration in supernatants was determined by BCA protein assay (Thermo Scientific) according to the manufacturer's instructions. Equal amounts of homogenates were loaded with reducing sample buffer. SC were lysed and boiled in 2X SDS dye lysis buffer (1 M Tris-HCl pH 6.8, 10% Sodium Dodecyl Sulfate, Glycerol, 1% Dichloro-Diphenyl-Tichloroethane, 1% Bromophonol blue) and centrifuged at 17,000 g at 4°C, the supernatants were denatured, resolved on SDS-polyacrylamide gel and electroblotted onto PVDF or nitrocellulose membrane (Millipore). Blots were then blocked with BSA 5% in PBS or Odyssey buffer (LI-COR Biosciences) and incubated with the appropriate antibody. Blots were developed with ECL or ECL prime (GE healthcare), and band intensity was quantified from films using ImageJ software. Alternatively, for quantitative WB analyses, filters were analyzed using the Odyssey Infrared Imaging System (LI-COR Biosciences) according to manufacturer's instructions.

### Immunohistochemistry

DRG cocultures were fixed with 4% PFA for 20 min, washed, permeabilized with cold methanol for 20 min, incubated in blocking solution (20% FCS, 2% bovine serum albumin, and 0.1% Triton in PBS) for at least 1 h, and then incubated overnight with antineurofilament, MBP, or PH3 antibodies in blocking solution. Explants were then incubated with secondary antibodies and counterstained with Dapi. 6 (for MBP) or 5 (for PH3) images from each DRG were acquired by epifluorescence on a Leica DM5500B or DM6000 microscope with a 10X or 20X objective. This analysis was performed on at least 3 coverslips per embryo and on 2 (for PH3) or 3 (for MBP) embryos per genotype for at least of 6 (for PH3) or 9 (for MBP) coverslips per condition. Sciatic nerves were dissected from P5 or P16 mice and fixed 1 h in 4% PFA at 4°C, cryo-protected in 20% sucrose (Sigma-Aldrich), embedded in OCT (Miles), and snap-frozen in liquid nitrogen. Alternatively, unfixed nerves were directly embedded in OCT and snap frozen. Staining was performed on 10-μm longitudinal sections on unfixed tissue for Egr2 and fixed tissue for Oct6. Sections were permeabilized in cold methanol for 5 min and blocked with 20% FBS, 2% BSA, and 0.1% triton and incubated overnight with the primary antibody. Images from 3 different sections per animal were acquired with a 20X objective. 3 different mice per for each genotype were analyzed.

### TUNEL and proliferation assay

These assays were performed as described [84].

### PKA Assay

PKA activity was assessed in vivo using the SigaTect cAMP-dependent PKA assay system (Promega, V4780). Sciatic nerves at P5 and P16 were sampled and immediately used for the assay according to the manufacturer instructions.

### cAMP Assay

To measure cAMP concentration in P5 and P16 sciatic nerves, a cAMP assay kit (Enzo Life Sciences) was used as described [85].

### In vivo injections

Treatments with the PKA signaling inhibitors H-89 (EMD Millipore) and KT5720 from (Enzo Life Sciences) were performed through injections beneath the gluteus superficialis and biceps femoris muscles. Animals were injected on 4 consecutive days from P3 to P6 with 10 μl of inhibitor solution (10 μM H-89 DMSO, 1 μM KT5720, 0.1% DMSO diluted PBS) per day. Untreated nerves were injected with 10 μl of 0.1% DMSO diluted in PBS. Sciatic nerves were sampled at P7.

### Statistical analyses

Data were collected randomly and assessed blindly. The data distribution was assumed to be normal, although we did not formally test it. All statistical analyses were performed on at least 3 independent experiments. Statistical detailed analyses are reported in each figure legend and all assays (1-way ANOVA multiple comparison test, 2-sided moderate *t* test and 2-tailed unpaired *t* test) were performed using the Prism Software package (GraphPad, San Diego, CA).

The numerical data used in all figures are included in S1 Data.

## Supporting information

S1 Data Excel spreadsheet containing, in separate sheets, the underlying numerical data for Figure panels 1M, 1N, 2C, 2D, 3B–3E, 4C, 5B–5E, 6B, 6E, 6F, 7A, 7B, 7D, 7E, 7G, 7H, 8B, 8D, 8E, S1C, S1D, S2A–S2D, S4A–S4D and S5A and S5B. (XLSX)

S1 Fig Defects in SC proliferation and death are not major causes of radial sorting defects in *Nrg1III*^*tg*^*//Lama2*^*−/−*^ mice. (A, B) Morphology of developing sciatic nerves from the indicated genotypes at P5. (A) Transverse semithin sections show that in P5 wild-type nerves, radial sorting is ongoing. *Nrg1III*^*tg*^*//Lama2*^*−/−*^ mice present more bundles of naked axons (arrowheads) and fewer myelinated axons than *Lama*2^−/−^ mice. (B) Electron micrograph analysis shows that in *Lama2*^*−/−*^ and *Nrg1III*^*tg*^*//Lama2*^*−/−*^ mutants, large caliber axons (a) are naked and grouped in bundles. One axon is undergoing axonal degeneration (arrow). (C, D) Longitudinal sciatic nerve sections of control and mutant mice at P5 (C) or P16 (D) were stained with P-H3 (green) or TUNEL (red) and counterstained with DAPI (blue). There are no statistically significant differences among the genotypes in the percentage of P-H3 nuclei positive or TUNEL-positive nuclei. n = 3 mice per genotype. Data are represented as mean value ± sem *p ≤ 0.05. Bar = 10 μm in A, 2 μm in B, 100 μm in C, D. The numerical data used in C and D are included in S1 Data.
(TIF)

S2 Fig AKT, Erk, cAMP and PKA activity in Lm211 and Nrg1II mutant nerves. (A) Western blot from P16 sciatic nerves shows that the activation of the Erk and Akt pathways is not significantly different among the mutants. The graphs on the right show quantification for *n* = 3 (Akt) and *n* = 2 (Erk) nerves. Error bars indicate SEM. (B) PKA activity in sciatic nerves from the mice of the indicated genotypes at P5. PKA is more active in *Lama*2^−/−^ and *Nrg1III*^*tg*^ sciatic nerves. (C, D) Measurement of cAMP from sciatic nerves at P5 and P16 from the indicated genotypes. cAMP is lower in *Lama*2^−/−^ sciatic nerves at P16. The numerical data used in A-D are included in S1 Data.
(TIF)

S3 Fig Summary of radial sorting and myelin phenotypes in the mutants. (A) Summary of radial sorting and myelin phenotypes observed in single and double mutants. (B) Schematic of Lm211 inhibiting pro-myelinating pathways downstream of Nrg1III by negatively regulating PKA (solid arrows). The dotted arrow shows the putative activation of PKA by Nrg1III, as indicated in the literature [42,43], which would result in inhibition of radial sorting by Nrg1III. In this view, the inhibition between Lm211 and Nrg1III would be reciprocal.
(TIF)

S4 Fig Egr2, Gab1, Oct6, and Egr2 at P5 in sciatic nerves from *Lama*2^−/−^ and *Nrg1III*^*tg*^//*Lama*2^−/−^ mutants. Western blot from P5 sciatic nerves for p-ErbB2 (A) p-Gab1 (B), Oct6 (C) and Egr2 (D) show a trend for increase in p-ErbB2, p-Gab1, and Egr2 in *Lama*2^−/−^ and *Nrg1III*^*tg*^//*Lama*2^−/−^ double mutant nerves, less evident when Nrg1III is overexpressed alone. The experiments were repeated 2 times from 2 different animals per genotype. Error bars indicate SEM. The numerical data used in A-D are included in S1 Data.
(TIF)

S5 Fig Short pharmacological treatment with PKA inhibitors in vivo does not reduce the expression of Oct6 or Egr2. (A, B) Intermuscular injection of HB9 and KT5720 for 4 days did not significantly reduce Oct6 (A) or Egr2 (B) expression in P7 sciatic nerves. The experiments were repeated at least 3 times on 3 animals per genotype. The numerical data used in A-B are included in S1 Data.
(TIF)

## Abbreviations

cAMP: 3′-5′-cyclic adenosine monophosphate
DLG1: Disc Large MAGUK scaffold protein 1
DRG: dorsal root ganglia
ECM: extracellular matrix
Egr2: early growth response protein 2
EM: electron microscopy
EPAC: exchange protein directly activated by cAMP
Gab1: Grb2-Associated Binder-1
Lm211: laminin α2β1γ1
MBP: myelin basic protein
Nrg1: neuregulin 1
Nrg1III: neuregulin 1 type III
P16: postnatal day 16
P-H3: phosphorylated-histone3
PKA: protein kinase A
PTEN: Phosphatase and Tensin homolog
SC: Schwann cell
WB: western blot
WT: wild-type
