# Supplementary figures and images for: Laminin 211 inhibits protein kinase A in Schwann cells to modulate neuregulin 1 type III-driven myelination
Source: PLoS Biol. 2017 Jun 21;15(6):e2001408. doi: 10.1371/journal.pbio.2001408 (PMC5479503; doi:10.1371/journal.pbio.2001408)

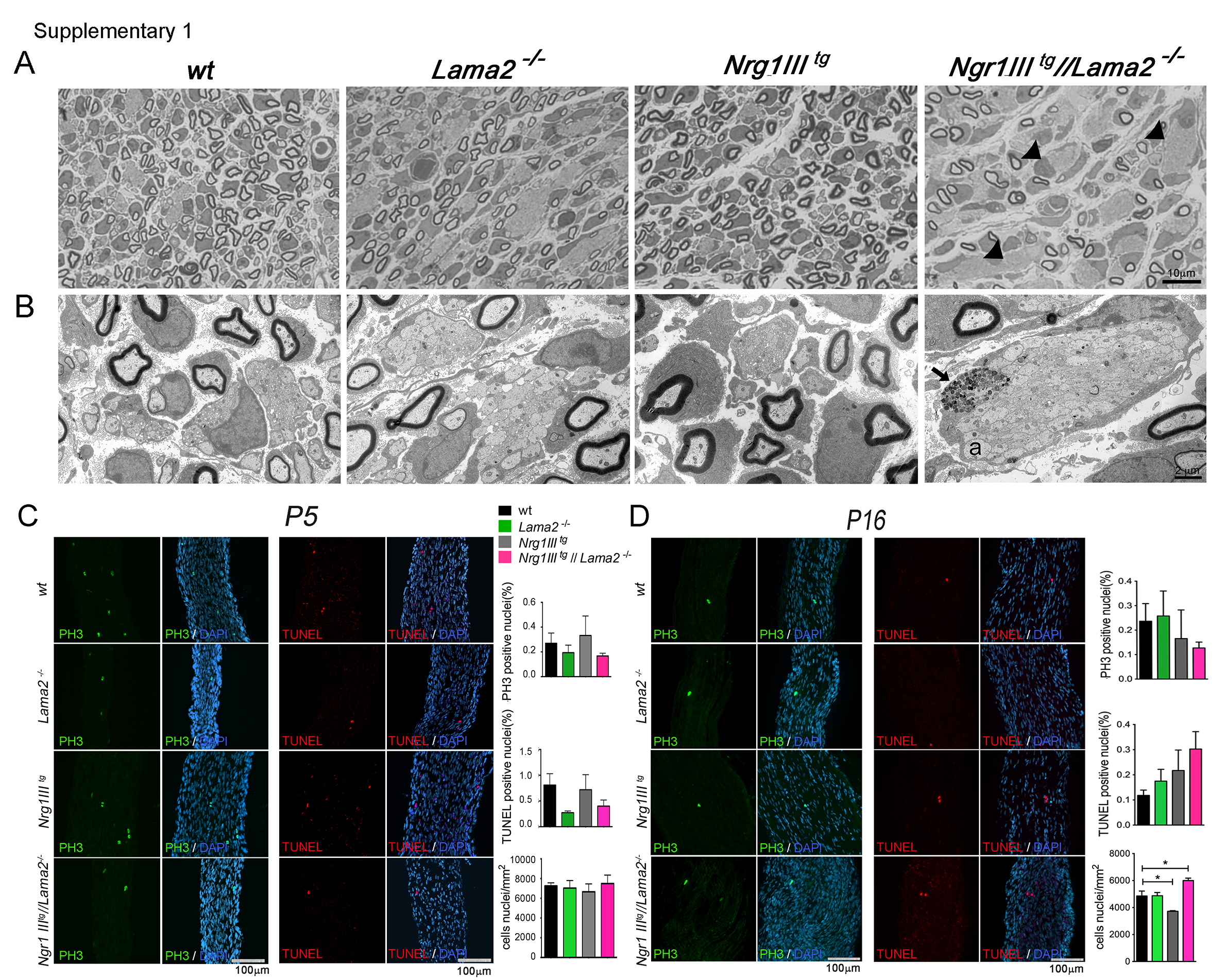

Supplement: S1 Fig — (A, B) Morphology of developing sciatic nerves from the indicated genotypes at P5. (A) Transverse semithin sections show that in P5 wild-type nerves, radial sorting is ongoing. Nrg1IIItg//Lama2−/− mice present more bundles of naked axons (arrowheads) and fewer myelinated axons than Lama2−/− mice. (B) Electron micrograph analysis shows that in Lama2−/− and Nrg1IIItg//Lama2−/− mutants, large caliber axons (a) are naked and grouped in bundles. One axon is undergoing axonal degeneration (arrow). (C, D) Longitudinal sciatic nerve sections of control and mutant mice at P5 (C) or P16 (D) were stained with P-H3 (green) or TUNEL (red) and counterstained with DAPI (blue). There are no statistically significant differences among the genotypes in the percentage of P-H3 nuclei positive or TUNEL-positive nuclei. n = 3 mice per genotype. Data are represented as mean value ± sem *p ≤ 0.05. Bar = 10 μm in A, 2 μm in B, 100 μm in C, D. The numerical data used in C and D are included in S1 Data. (TIF) [file pbio.2001408.s002.tif]

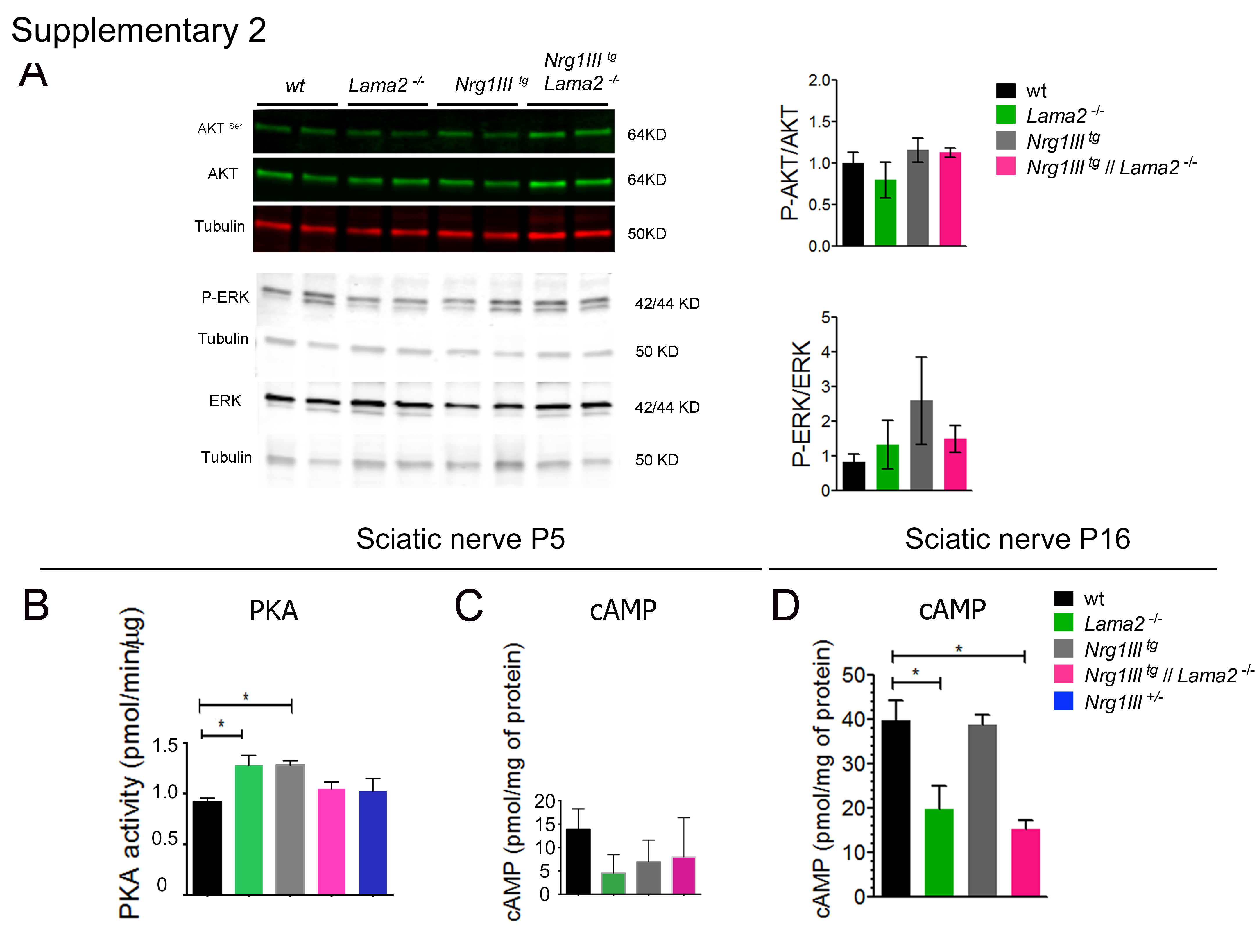

Supplement: S2 Fig — (A) Western blot from P16 sciatic nerves shows that the activation of the Erk and Akt pathways is not significantly different among the mutants. The graphs on the right show quantification for n = 3 (Akt) and n = 2 (Erk) nerves. Error bars indicate SEM. (B) PKA activity in sciatic nerves from the mice of the indicated genotypes at P5. PKA is more active in Lama2−/− and Nrg1IIItg sciatic nerves. (C, D) Measurement of cAMP from sciatic nerves at P5 and P16 from the indicated genotypes. cAMP is lower in Lama2−/− sciatic nerves at P16. The numerical data used in A-D are included in S1 Data. (TIF) [file pbio.2001408.s003.tif]

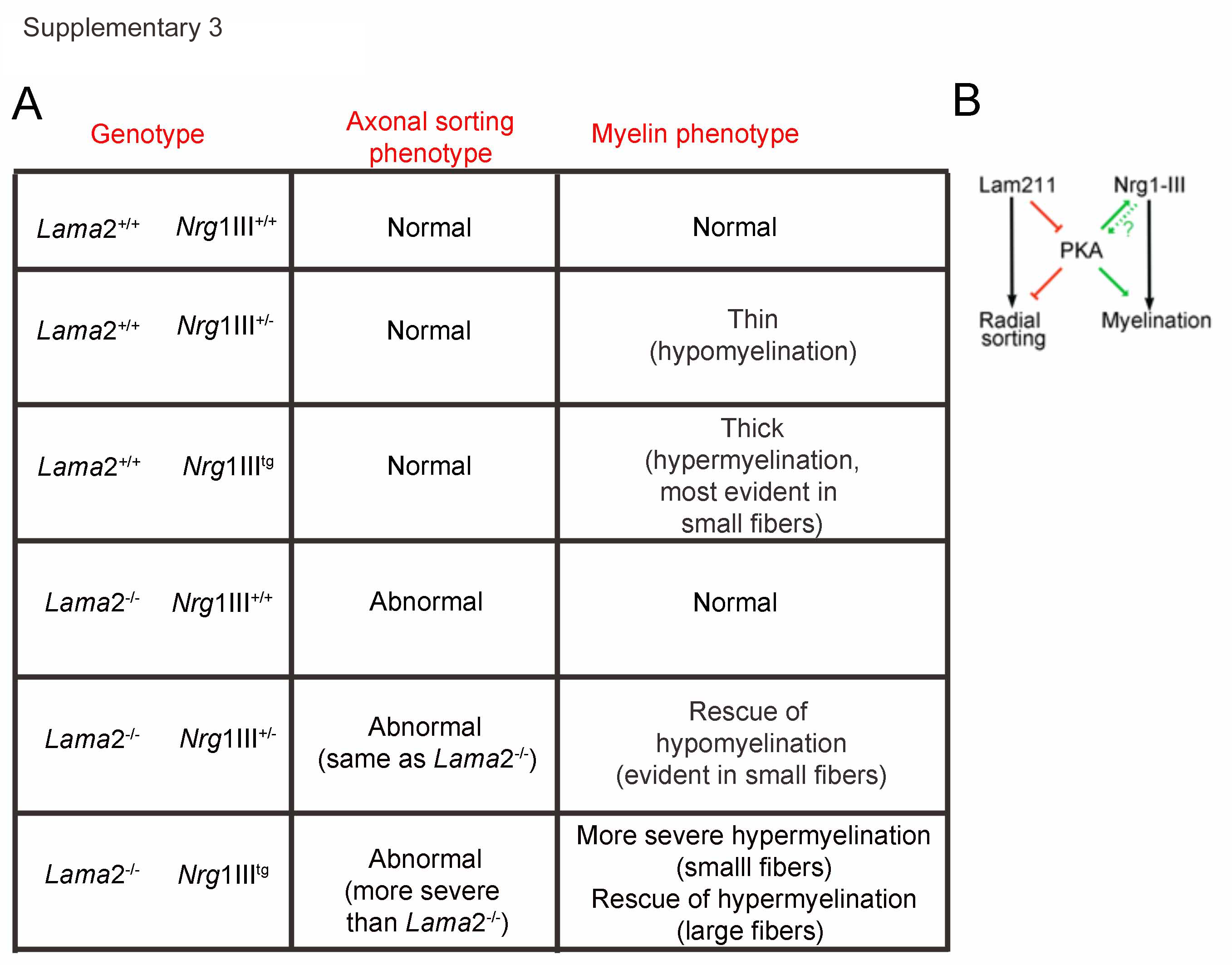

Supplement: S3 Fig — (A) Summary of radial sorting and myelin phenotypes observed in single and double mutants. (B) Schematic of Lm211 inhibiting pro-myelinating pathways downstream of Nrg1III by negatively regulating PKA (solid arrows). The dotted arrow shows the putative activation of PKA by Nrg1III, as indicated in the literature [42,43], which would result in inhibition of radial sorting by Nrg1III. In this view, the inhibition between Lm211 and Nrg1III would be reciprocal. (TIF) [file pbio.2001408.s004.tif]

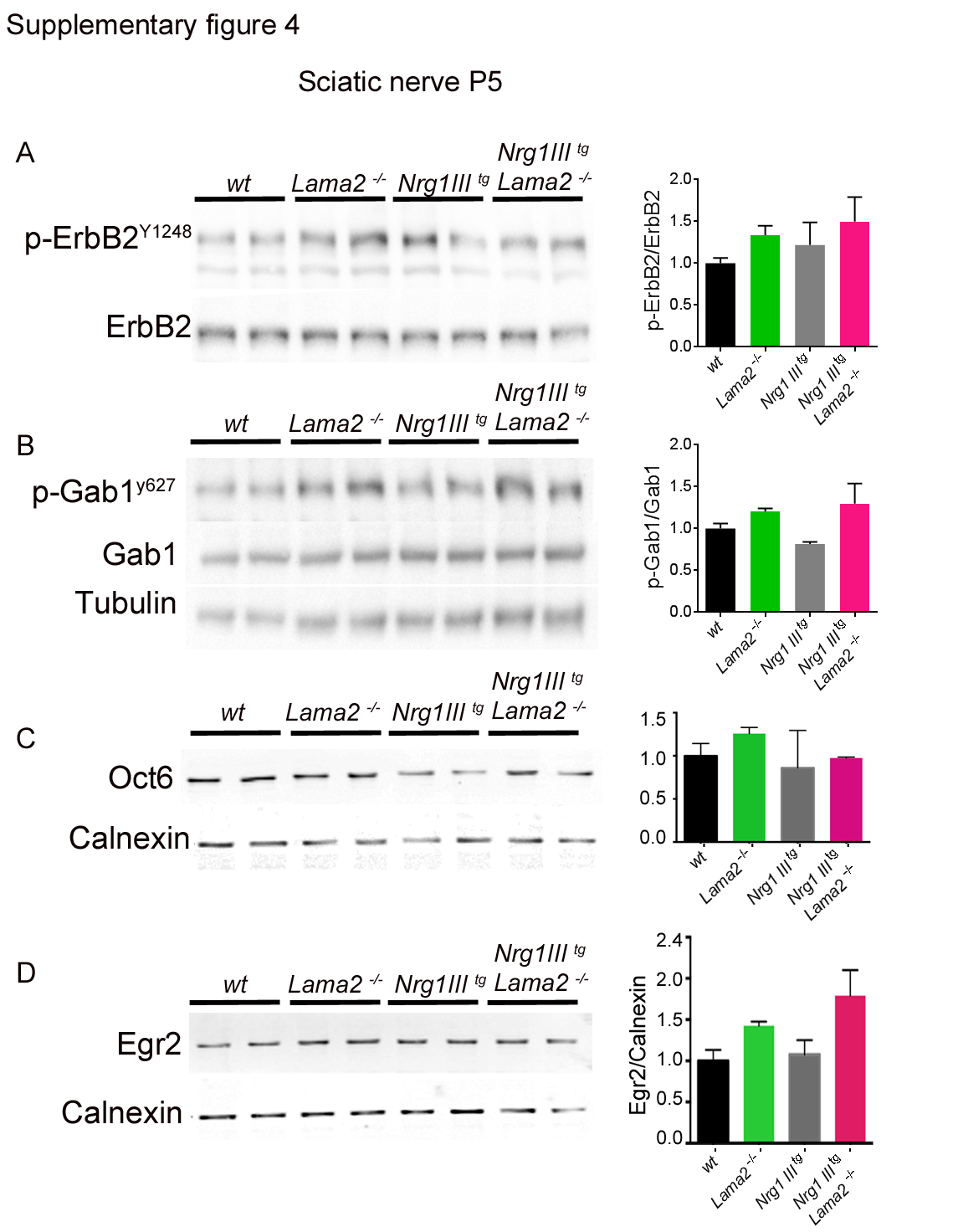

Supplement: S4 Fig — Western blot from P5 sciatic nerves for p-ErbB2 (A) p-Gab1 (B), Oct6 (C) and Egr2 (D) show a trend for increase in p-ErbB2, p-Gab1, and Egr2 in Lama2−/− and Nrg1IIItg//Lama2−/− double mutant nerves, less evident when Nrg1III is overexpressed alone. The experiments were repeated 2 times from 2 different animals per genotype. Error bars indicate SEM. The numerical data used in A-D are included in S1 Data. (TIF) [file pbio.2001408.s005.tif]

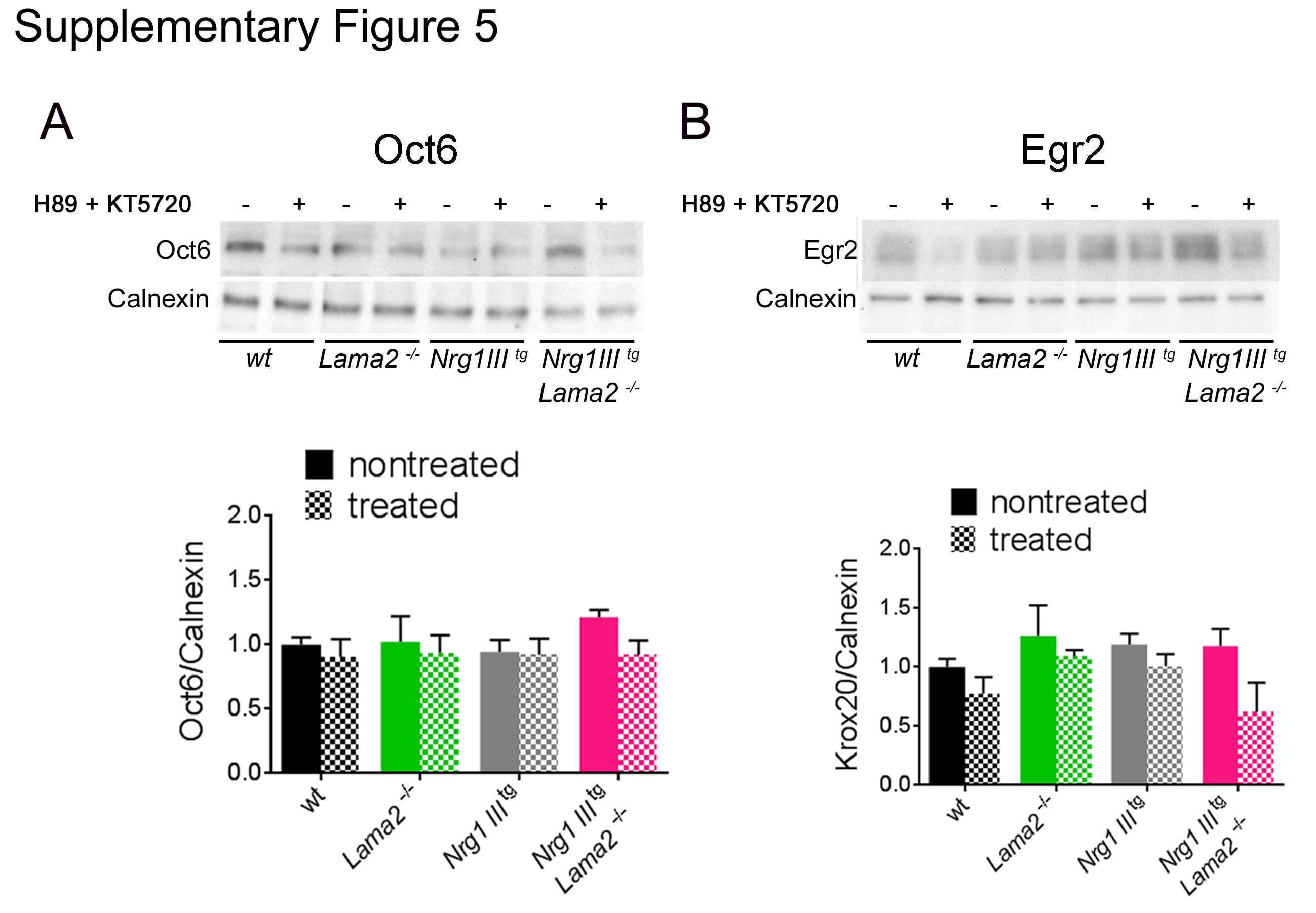

Supplement: S5 Fig — (A, B) Intermuscular injection of HB9 and KT5720 for 4 days did not significantly reduce Oct6 (A) or Egr2 (B) expression in P7 sciatic nerves. The experiments were repeated at least 3 times on 3 animals per genotype. The numerical data used in A-B are included in S1 Data. (TIF) [file pbio.2001408.s006.tif]
